# Canadian clinical practice guidelines for acute and chronic rhinosinusitis

**DOI:** 10.1186/1710-1492-7-2

**Published:** 2011-02-10

**Authors:** Martin Desrosiers, Gerald A Evans, Paul K Keith, Erin D Wright, Alan Kaplan, Jacques Bouchard, Anthony Ciavarella, Patrick W Doyle, Amin R Javer, Eric S Leith, Atreyi Mukherji, R Robert Schellenberg, Peter Small, Ian J Witterick

**Affiliations:** 1 Division of Otolaryngology - Head and Neck Surgery Centre Hospitalier de l'Université de Montréal, Université de Montréal Hotel-Dieu de Montreal, and Department of Otolaryngology - Head and Neck Surgery and Allergy, Montreal General Hospital, McGill University, Montreal, QC, Canada; 2 Division of Infectious Diseases, Department of Medicine, and Departments of Microbiology & Immunology and Pathology & Molecular Medicine, Queen's University and Kingston General Hospital, Kingston, ON, Canada; 3 Deparmtent of Medicine, Division of Allergy and Clinical Immunology, McMaster University Hamilton, ON, Canada; 4 Division of Otolaryngology - Head and Neck Surgery, University of Alberta, Edmonton, AB, Canada; 5 Family Physician Airways Group of Canada and Brampton Civic Hospital, Richmond Hill, ON, Canada; 6 Clinical Medicine, Laval's University Quebec and Department of Medicine, Hôpital de la Malbaie La Malbaie, QC, Canada; 7 Family Physician Airways Group of Canada, Aldergrove, BC, Canada; 8 Division of Medical Microbiology and Infection Control, Vancouver General Hospital and Department of Pathology and Laboratory Medicine, University of British Columbia, Vancouver, BC, Canada; 9 St Paul's Sinus Center and Division of Otolaryngology-Head and Neck Surgery, University of British Columbia, Vancouver, BC, Canada; 10 Department of Medicine, University of Toronto and Women's College Hospital, Halton Healthcare Services (Oakville Trafalger Site), Toronto, ON, Canada; 11 Department of Medicine, Division of Infectious Diseases, McMaster University and Department of Medicine/Infectious Diseases, Hamilton General Hospital, McMaster Wing Hamilton, ON, Canada; 12 Department of Medicine, Division of Allergy and Immunology, University of British Columbia, and James Hogg iCAPTURE Centre for Cardiovascular and Pulmonary Research, St. Paul's Hospital Vancouver, BC, Canada; 13 Division of Allergy and Clinical Immunology, Jewish General Hospital and Department of Medicine, Department of Medicine, McGill University, Montreal, QC, Canada; 14 Department of Otolaryngology-Head & Neck Surgery, University of Toronto, Toronto, ON, Canada

## Abstract

This document provides healthcare practitioners with information regarding the management of acute rhinosinusitis (ARS) and chronic rhinosinusitis (CRS) to enable them to better meet the needs of this patient population. These guidelines describe controversies in the management of acute bacterial rhinosinusitis (ABRS) and include recommendations that take into account changes in the bacteriologic landscape. Recent guidelines in ABRS have been released by American and European groups as recently as 2007, but these are either limited in their coverage of the subject of CRS, do not follow an evidence-based strategy, or omit relevant stakeholders in guidelines development, and do not address the particulars of the Canadian healthcare environment.

Advances in understanding the pathophysiology of CRS, along with the development of appropriate therapeutic strategies, have improved outcomes for patients with CRS. CRS now affects large numbers of patients globally and primary care practitioners are confronted by this disease on a daily basis. Although initially considered a chronic bacterial infection, CRS is now recognized as having multiple distinct components (eg, infection, inflammation), which have led to changes in therapeutic approaches (eg, increased use of corticosteroids). The role of bacteria in the persistence of chronic infections, and the roles of surgical and medical management are evolving. Although evidence is limited, guidance for managing patients with CRS would help practitioners less experienced in this area offer rational care. It is no longer reasonable to manage CRS as a prolonged version of ARS, but rather, specific therapeutic strategies adapted to pathogenesis must be developed and diffused.

Guidelines must take into account all available evidence and incorporate these in an unbiased fashion into management recommendations based on the quality of evidence, therapeutic benefit, and risks incurred. This document is focused on readability rather than completeness, yet covers relevant information, offers summaries of areas where considerable evidence exists, and provides recommendations with an assessment of strength of the evidence base and degree of endorsement by the multidisciplinary expert group preparing the document.

These guidelines have been copublished in both *Allergy, Asthma & Clinical Immunology *and the *Journal of Otolaryngology-Head and Neck Surgery*.

## Introduction

Sinusitis refers to inflammation of a sinus, while rhinitis is inflammation of the nasal mucous membrane. The proximity between the sinus cavities and the nasal passages, as well as their common respiratory epithelium, lead to frequent simultaneous involvement of both structures (such as with viral infections). Given the difficulty separating the contributions of deep structure to signs and symptoms, the term rhinosinusitis is frequently used to describe this simultaneous involvement, and will be used in this text. Rhinosinusitis refers to inflammation of the nasal cavities and sinuses. When the inflammation is due to bacterial infection, it is called bacterial rhinosinusitis.

Rhinosinusitis is a frequently occurring disease, with significant impact on quality of life and health care spending, and economic impact in terms of absenteeism and productivity. It is estimated that approximately 6 billion dollars is spent in the United States annually on therapy for rhinosinusitis [1]. A recent study in Canada described the impact of chronic rhinosinusitis (CRS) on patients and healthcare utilization [2]. Patients with CRS had a health status similar to patients with arthritis, cancer, asthma, and inflammatory bowel disease. Compared with people without CRS, those with CRS reported more days spent bedridden and more visits to family physicians, alternative healthcare providers, and mental health experts. These findings underscore the significant impact of this disease on patient quality of life, as well as costs of care to patients and society.

In Canada, 2.89 million prescriptions were dispensed for acute rhinosinusitis (ARS) or CRS in 2006, with approximately 2/3 for ARS and 1/3 for CRS [3]. Despite well-established differences between these 2 diseases in pathophysiology, bacteriology, and standard specialist treatment strategies, an assessment of therapies prescribed in Canada for CRS has shown that medications prescribed for CRS exactly paralleled those prescribed for ARS [3].

The incidence of bacterial rhinosinusitis is difficult to obtain precisely given that not all patients will seek medical help. In the United States in 2007, ARS affected 26 million individuals and was responsible for 12.9 million office visits [4]. Although no specific Canadian data is available, extrapolation from US data suggests an occurrence of 2.6 million cases in Canada annually. This is in line with prescription data from 2004. This high incidence is not unexpected given that acute bacterial rhinosinusitis (ABRS) usually develops as a complication in 0.5%-2% of upper respiratory tract infections (URTIs) [5].

A survey of Canadian households reported the prevalence of CRS to be 5% [6]. The prevalence was higher in women compared with men (5.7% vs 3.4% for subjects aged ≥12 years) and increased with age. CRS was associated with smoking, lower income, history of allergy, asthma, or chronic obstructive pulmonary disease (COPD), and was slightly higher for those living in the eastern region or among native Canadians.

Guidelines for ARS have been developed over the past 5 years by both a European group (E3POS) and the American Academy of Otolaryngology-Head and Neck Surgery (AAO-HNS). Both guidelines have limitations that we believe are improved upon by the current document. This current document provides healthcare practitioners with a brief, easy-to-read review of information regarding the management of ARS and CRS. These guidelines are meant to have a practical focus, directed at first-line practitioners with an emphasis on patient-centric issues. The readership is considered to be family physicians, emergency physicians, or other point-of-care providers, as well as specialists in otolaryngology-head and neck surgery, allergy and immunology, or infectious disease who dispense first-line care or teach colleagues on the subject. This document is specifically adapted for the needs of the Canadian practice environment and makes recommendations that take into account factors such as wait times for computed tomography scans or specialist referral. These guidelines are intended to provide useful information for CRS by addressing this area where controversy is unresolved and evidence is typically Grade D - requiring incorporation of expert opinion based on pathophysiology and current treatment regimens. Thus, the main thrust is to provide a comprehensive guide to CRS and to address changes in the management of ABRS.

## Guideline Preparation Process

An increased emphasis on evidence-based recommendations over the past decade has significantly improved the overall quality of most published guidelines, but present significant difficulties in developing guidelines where the evidence base for long-standing, traditional remedies is often weak or anecdotal, or in emerging entities such as chronic rhinosinusitis (CRS) where controversy remains and evidence is sparse. In developing these guidelines, standard evidence-based development techniques have been combined with the Delphi voting process in order to offer the reader the opinion of a multidisciplinary expert group in areas where evidence is weak.

Funding was obtained via an unrestricted grant obtained from 5 pharmaceutical manufacturers, with each contributing equally to this project. In order to minimize any appearance of conflict of interest, all funds were administered via a trust account held at the Canadian Society of Otolaryngology-Head and Neck Surgery (CSO-HNS). No contact with industry was made during the guidelines development or review process.

An English-language Medline^® ^search was conducted using the terms acute bacterial rhinosinusitis (ABRS), chronic rhinosinusitis (CRS), and nasal polyposis (limited to the adult population, human, clinical trials, items with abstracts) and further refined based on the individual topics. This is a multi-disciplined condition and therefore input from all appropriate associations was required. Inclusion criteria: most current evidence-based data, relevance, subject specifics, caliber of the abstract, Canadian data preferred but not exclusive. Exclusion criteria: newer abstract of the same subject available, non-human, not relevant.

The quality of retrieved articles was assessed by Society Team Leaders along with the principal author based on area of expertise. Where necessary, the principal author invited input from the External Content Experts. Articles were graded for strength of evidence by drawing upon strategies adapted from the American Academy of Pediatrics Steering Committee on Quality Improvement and Management (AAP SCQIM) guidelines [7], the Grades of Recommendation, Assessment, Development and Evaluation (GRADE) grading system [8], and the AAO-HNS guidelines in sinusitis [9], all of which use similar strategies by classifying strength of evidence recommendations according to the balance of the benefits and downsides after considering the quality of the evidence. Accordingly, grades of evidence were defined as:

Grade A. Well-designed, randomized, controlled studies or diagnostic studies on relevant populations

Grade B. Randomized controlled trials or diagnostic studies with minor limitations; overwhelmingly consistent evidence from observational studies

Grade C. Observational studies (case control or cohort design)

Grade D. Expert opinion, case reports, reasoning from first principles

Grade X. Exceptional situations where validating studies cannot be done and there is a clear predominance of benefit or harm [7].

### Strength of Evidence

Definitions for the strength of evidence recommendations combine the balance of benefit versus harm of treatment with the grade of the evidence, as follows:

*Strong Recommendation*: Benefits of treatment clearly exceed harm; quality of evidence is excellent (Grade A or B). A strong recommendation should be followed unless there is a clear and compelling reason for a different approach.

*Recommendation*: Benefits exceeded harm, but quality of evidence is not as strong (Grade B or C). A recommendation should generally be followed, but clinicians should remain alert to new information and consider patient preferences.

*Option*: Quality of evidence is suspect (Grade D) or well-done studies (Grade A, B or C) show little clear advantage. An option reflects flexibility in decision-making regarding appropriate practice, but clinicians may set limits on alternatives. The preference of the patient should influence the decision.

*No Recommendation*: A lack of relevant evidence (Grade D) and an unclear balance between benefits and harm. No recommendation reflects no limitations on decision-making and clinicians should be vigilant regarding new information on the balance of benefit versus harm. The preference of the patient should influence the decision.

In situations where high-quality evidence is impossible to obtain and anticipated benefits strongly outweigh the harm, the recommendation may be based on lesser evidence [9].

Thus, policy recommendations were formulated based on evidence quality and the balance of potential benefits and harm. As many therapies have not been subjected to safety evaluation in a clinical trial setting, the potential for harm was assessed for each therapy and weighs in the recommendation. The guidelines presented used these approaches to formulate strength of evidence recommendations, with options to recommend denoted as:

• Strong

• Moderate

• Weak

• An option for therapy, or

• Not recommended as either clinical trial data of a given therapy did not support its use or a concern for toxicity was noted.

### Strength of Recommendation

Recommendations were assessed according to a Delphi voting process, whereby voting options included to accept completely, to accept with some reservation, to accept with major reservation, to reject with reservation, or to reject completely [7,10]. Only statements that were accepted by over 50% of the group were retained. Strength of the recommendation by the multidisciplinary group of experts was denoted as:

• Strong (for accept completely)

• Moderate (for accept with some reservation), or

• Weak (for accept with major reservation).

Thus, strength of recommendation is a measure of endorsement by the group of experts.

These guidelines have been developed from the outset to meet the AGREE criteria [11] to ensure maximum impact.

DISCLAIMER: These guidelines are designed to offer evidence-based strategies in the management of acute and chronic rhinosinusitis. They are, however, not intended to replace clinical judgment or establish a protocol for all individuals with suspected rhinosinusitis. Different presentations, associated comorbidities, or availability of resources may require adaptation of these guidelines, thus there may be other appropriate approaches to diagnosing and managing these conditions.

## Summary of Guideline Statements and Strengths

Statements and their ratings for strength of evidence and recommendation are summarized in Table 1.

**Table 1:** Guideline Statements and Strengths for Acute Bacterial Rhinosinusitis and Chronic Rhinosinusitis

Statement	Strength of Evidence*	**Strength of Recommendation**^**†**^
**Acute Bacterial Rhinosinusitis**		

**1**: ABRS may be **diagnosed **on clinical grounds using symptoms and signs of more than 7 days duration.	Moderate	Strong

**2**. Determination of **symptom severity **is useful for the management of acute sinusitis, and can be based upon the intensity and duration and impact on patient's quality of life.	Option	Strong

**3: Radiological imaging **is not required for the diagnosis of uncomplicated ABRS. When performed, radiological imaging must always be interpreted in light of clinical findings as radiographic images cannot differentiate other infections from bacterial infection and changes in radiographic images can occur in viral URTIs.	Moderate	Strong
**Criteria for diagnosis **of ABRS are presence of an air/fluid level or complete opacification. Mucosal thickening alone is not considered diagnostic. Three-view plain sinus X-rays remain the standard. Computed tomography (CT) scanning is mainly used to assess potential complications or where regular sinus X-rays are no longer available.		
**Radiology **should be considered to confirm a diagnosis of ARBS in patients with multiple recurrent episodes, or to eliminate other causes.		

**4: Urgent consultation **should be obtained for acute sinusitis with unusually severe symptoms *or *systemic toxicity *or *where orbital or intracranial involvement is suspected.	Option	Strong

**5: Routine nasal culture **is not recommended for the diagnosis of ABRS. When culture is required for unusual evolution, or when complication requires it, sampling must be performed either by maxillary tap or endoscopically-guided culture.	Moderate	Strong

**6**: The 2 main **causative infectious bacteria **implicated in ABRS are *Streptococcus pneumoniae *and *Haemophilus influenzae.*	Strong	Strong

**7: Antibiotics **may be prescribed for ABRS to improve rates of resolution at 14 days and should be considered where either quality of life or productivity present as issues, or in individuals with severe sinusitis or comorbidities. In individuals with mild or moderate symptoms of ABRS, if quality of life is not an issue and neither severity criterion nor comorbidities exist, antibiotic therapy can be withheld.	Moderate	Moderate

**8**: When **antibiotic therapy **is selected, amoxicillin is the first-line recommendation in treatment of ABRS. In beta-lactam allergic patients, trimethoprim-sulfamethoxazole (TMP/SMX) combinations or a macrolide antibiotic may be substituted.	Option	Strong

**9: Second-line therapy **using amoxicillin/clavulanic acid combinations or quinolones with enhanced gram positive activity should be used in patients where risk of bacterial resistance is high, or where consequences of failure of therapy are greatest, as well as in those not responding to first-line therapy. A careful history to assess likelihood of resistance should be obtained, and should include exposure to antibiotics in the prior 3 months, exposure to daycare, and chronic symptoms.	Option	Strong

**10: Bacterial resistance **should be considered when selecting therapy.	Strong	Strong

**11**: When antibiotics are prescribed, **duration of treatment **should be 5 to 10 days as recommended by product monographs. Ultra-short treatment durations are not currently recommended by this group.	Strong	Moderate

**12**: Topical **intranasal corticosteroids **(INCS) can be useful as sole therapy of mild-to-moderate ARS.	Moderate	Strong

**13: Treatment failure **should be considered when patients fail to respond to initial therapy within 72 hours of administration. If failure occurs following use of INCS as monotherapy, antibacterial therapy should be administered. If failure occurs following antibiotic administration, it may be due to lack of sensitivity to, or bacterial resistance to, the antibiotic, and the antibiotic class should be changed.	Option	Strong

**14: Adjunct therapy **should be prescribed in individuals with ABRS.	Option	Strong

**15**. Topical **INCS **may help improve resolution rates and improve symptoms when prescribed with an antibiotic.	Moderate	Strong

**16. Analgesics **(acetaminophen or non-steriodal anti-inflammatory agents) may provide symptom relief.	Moderate	Strong

**17. Oral decongestants **may provide symptom relief.	Option	Moderate

**18. Topical decongestants **may provide symptom relief.	Option	Moderate

**19. Saline irrigation **may provide symptom relief.	Option	Strong

**20**. For those not responding to a second course of therapy, **chronicity **should be considered and the patient referred to a specialist. If waiting time for specialty referral or CT exceeds 6 weeks, CT should be ordered and empiric therapy for CRS administered. Repeated bouts of acute uncomplicated sinusitis clearing between episodes require only investigation and referral, with a possible trial of INCS. Persistent symptoms of greater than mild-to-moderate symptom severity should prompt urgent referral.	Option	Moderate

**21**: By reducing transmission of respiratory viruses, hand washing can **reduce the incidence **of viral and bacterial sinusitis. Vaccines and prophylactic antibiotic therapy are of no benefit.	Moderate	Strong

**22: Allergy testing **or in-depth assessment of immune function is not required for isolated episodes but may be of benefit in identifying contributing factors in individuals with recurrent episodes or chronic symptoms of rhinosinusitis.	Moderate	Strong

**Chronic Rhinosinusitis**		

**23**: CRS is **diagnosed **on clinical grounds but must be confirmed with at least 1 objective finding on endoscopy or computed tomography (CT) scan.	Weak	Strong

**24: Visual rhinoscopy **assessments are useful in discerning clinical signs and symptoms of CRS.	Moderate	Moderate

**25**: In the few situations when deemed necessary, **bacterial cultures **in CRS should be performed either via endoscopic culture of the middle meatus or maxillary tap but not by simple nasal swab.	Option	Strong

**26**: The preferred means of **radiological imaging **of the sinuses in CRS is the CT scan, preferably in the coronal view. Imaging should always be interpreted in the context of clinical symptomatology because there is a high false-positive rate.	Moderate	Strong

**27**: CRS is an **inflammatory disease **of unclear origin where bacterial colonization may contribute to pathogenesis. The relative roles of initiating events, environmental factors, and host susceptibility factors are all currently unknown.	Weak	Moderate

**28: Bacteriology **of CRS is different from that of ABRS.	Moderate	Strong

**29**: Environmental and physiologic factors can **predispose **to development or recurrence of chronic sinus disease. Gastroesophageal reflux disease (GERD) has not been shown to play a role in adults.	Moderate	Strong

**30**: When diagnosis of CRS is suggested by history and objective findings, **oral or topical steroids with or without antibiotics **should be used for management.	Moderate	Moderate

**31**: Many **adjunct therapies **commonly used in CRS have limited evidence to support their use. **Saline irrigation **is an approach that has consistent evidence of benefiting symptoms of CRS.	Moderate	Moderate

**32**. Use of **mucolytics **is an approach that may benefit symptoms of CRS.	Option	Moderate

**33**. Use of **antihistamines **is an approach that may benefit symptoms of CRS.	Option	Weak

**34**. Use of **decongestants **is an approach that may benefit symptoms of CRS.	Option	Weak

**35**. Use of **leukotriene modifiers **is an approach that may benefit symptoms of CRS.	Weak	Weak

**36: Failure of response **should lead to consideration of other possible contributing diagnoses such as migraine or temporomandibular joint dysfunction (TMD).	Option	Moderate

**37: Surgery **is beneficial and indicated for individuals failing medical treatment.	Weak	Moderate

**38**: Continued use of medical **therapy post-surgery **is key to success and is required for all patients. Evidence remains limited.	Moderate	Moderate

**39 Part A**: Patients should be **referred **by their primary care physician when failing 1 or more courses of maximal medical therapy or for more than 3 sinus infections per year.	Weak	Moderate

**39 Part B: Urgent consultation **with the otolaryngologist should be obtained for individuals with severe symptoms of pain or swelling of the sinus areas or in immunosuppressed patients.	Weak	Strong

**40: Allergy testing **is recommended for individuals with CRS as potential allergens may be in their environment.	Option	Moderate

**41**: Assessment of **immune function **is not required in uncomplicated cases.	Weak	Strong

**42: Prevention **measures should be discussed with patients.	Weak	Strong

*Strength of evidence integrates the grade of evidence with the potential for benefit and harm.

^**†**^Strength of recommendation indicates the level of endorsement of the statement by the panel of experts.

## Acute Bacterial Rhinosinusitis (ABRS)

### Definition and Diagnosis

**Statement 1**: ABRS may be diagnosed on clinical grounds using symptoms and signs of more than 7 days duration.

**Strength of evidence**: Moderate

**Strength of recommendation**: Strong

**Rationale**: ABRS is a clinical diagnosis that must be differentiated from uncomplicated viral infections of the upper respiratory passages. Although no single symptom accurately predicts the presence or absence of bacterial infection, the presence of several signs and symptoms increases the predictive value.

#### Definition

The common cold is caused by a rhinovirus, and in most cases peak symptom severity is reached by 3 days [12]. However, the same virus can activate an inflammatory process that can lead to bronchitis, pharyngitis, and rhinosinusitis [13]. Thus, the term rhinosinusitis has been used to distinguish this more severe phenotypic entity from the common cold, which is associated with sinusitis [14]. Despite the frequency of the common cold, 0.5% to 2% of individuals with the common cold will develop ABRS [5].

ABRS is defined as a bacterial infection of the paranasal sinuses, described as a sudden onset of symptomatic sinus infection. Each episode usually lasts less than 4 weeks. Within this 4-week period, symptoms resolve either spontaneously or with appropriate treatment [15,16]. There may be up to 3 episodes per year and full recovery in between episodes. ABRS commonly occurs as a complication of a viral upper respiratory tract infection (URTI) [16,17] and is therefore difficult to differentiate from a viral infection. Recurrent ABRS is defined as 4 or more episodes of ABRS per year. Symptoms of ABRS have been classified as major and minor (Table 2) [18]. Although minor symptoms may be clinically helpful, they are not used for the diagnosis of ABRS.

**Table 2:** Symptoms of ABRS

Major	Minor
Facial pain/pressure/fullness	Headache
Nasal obstruction	Halitosis
Nasal purulence/discolored postnasal discharge	Fatigue
Hyposmia/anosmia	Dental pain
	Cough
	Ear pain/pressure

#### Diagnosis

**Figure 1:**
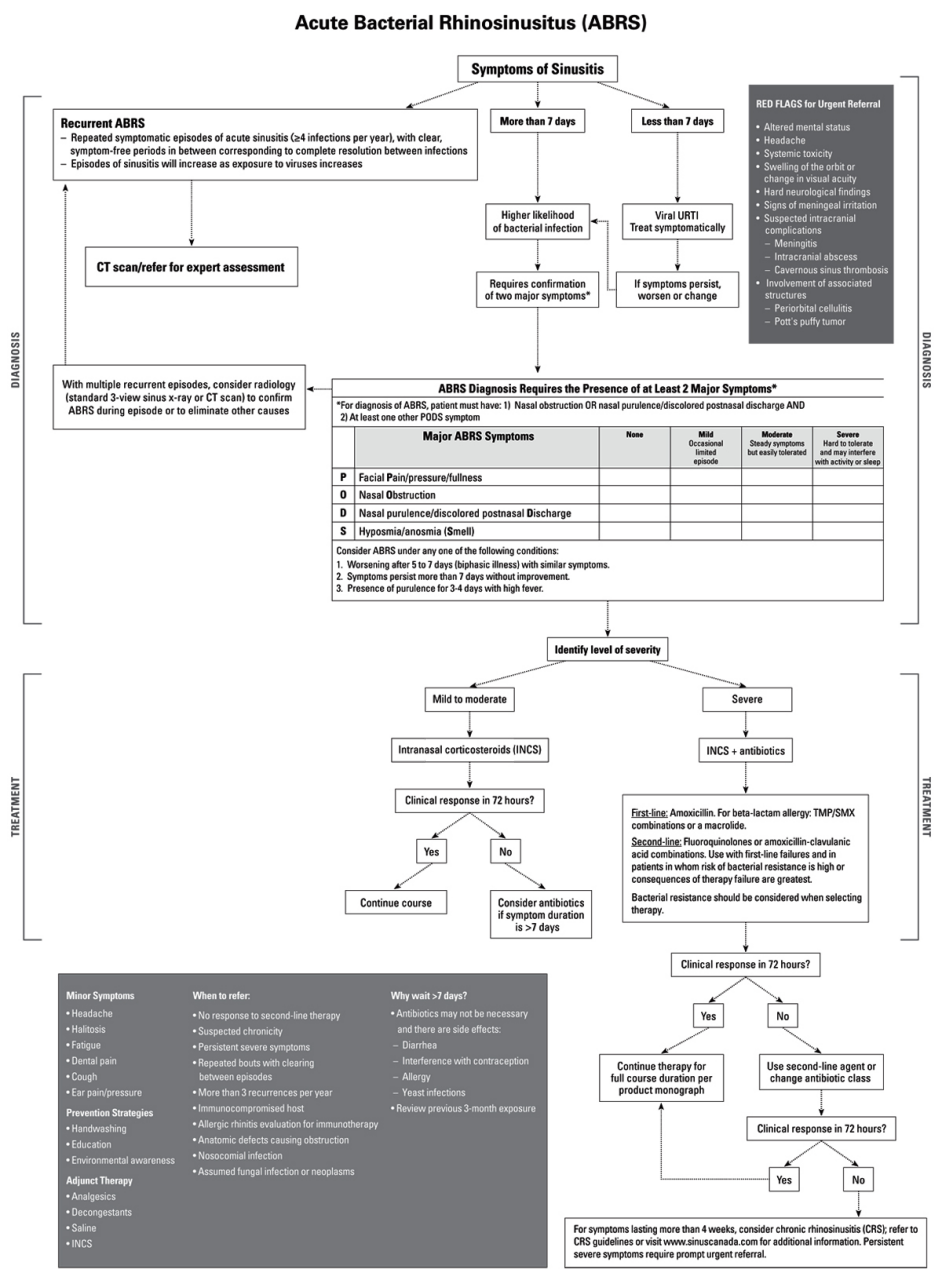
**Algorithm for the Diagnosis and Treatment of ABRS**.

Although sinus aspirates are considered to be the gold standard for diagnosis, this invasive procedure is not recommended in a primary care setting [15]. Clinicians thus must rely on history and physical examination for the initial evaluation of ABRS. ABRS can be diagnosed based on the presence of persistent or worsening symptoms (Table 3) [9,19-21]. An algorithm for the diagnosis and treatment of ABRS is presented in Figure 1.

**Table 3:** ABRS Diagnosis Requires the Presence of at Least 2 Major Symptoms*

	Major Symptom
**P**	Facial **P**ain/pressure/fullness

**O**	Nasal **O**bstruction

**D**	Nasal purulence/discolored postnasal **D**ischarge

**S**	Hyposmia/anosmia (**S**mell)

*At least 1 symptom must be nasal obstruction or nasal purulence/discolored postnasal discharge. Thus, a diagnosis requires at least 2 PODS, one of which must be O or D.

Consider ABRS when viral URTI persists beyond 10 days or worsens after 5 to 7 days with similar symptoms [22]. Bacterial etiology should be suspected if sinus symptoms persist for more than 7 days without improvement [20].

In sinus aspirate studies, symptoms lasting longer than 10 days were more likely due to ABRS [23]. The 7-to-10-day specification is based on the natural history of rhinovirus infections [22]. The presence of several signs and symptoms increases the predictive value.

Several consensus-based diagnostic criterion have been developed to aid clinicians in the diagnosis. The Centers for Disease Control and Prevention recommends reserving the diagnosis of ABRS for patients with:

• Symptoms lasting at least 7 days *and*

• Purulent nasal secretions *and*

• 1 of the following:

○ Maxillary pain

○ Tenderness in the face (especially unilateral)

○ Tenderness of the teeth (especially unilateral) [20].

Two studies of patients presenting with symptoms of sinusitis have led to the development of prediction rules. In 1 study, Berg et al reported that 2 or more positive findings provided 95% sensitivity and 77% specificity for sinusitis (Table 4) [24]. In the second study, Williams et al identified 5 independent predictors of sinusitis that were consistent with radiographic findings (Table 5) [25].

**Table 4:** Berg Prediction Rule Based on Signs and Symptoms of ABRS [24]

Sign or Symptom	Positive Predictive Value (PPV), %
Purulent rhinorrhea with unilateral predominance	50
Local pain with unilateral predominance	41
Pus in nasal cavity	17
Bilateral purulent rhinorrhea	15

Presence of ≥3 symptoms has a positive likelihood ratio (LR) of 6.75.

**Table 5:** Williams Prediction Rule Based on Signs and Symptoms of ABRS [25]

Sign or Symptom	Likelihood Ratio (LR) (present)
Maxillary toothache	2.5

Poor response to antihistamines/decongestants	2.1

Purulent nasal secretions	2.1

Abnormal transillumination	1.6

Colored nasal discharge	1.5

Presence of ≥4 symptoms has a positive LR of 6.4.

Prediction rules can be used to aid in diagnosis. Using either the Berg or Williams prediction rules, the probability of ABRS increases with cumulative symptoms [24,25]. Although none of these symptoms are individually sensitive or specific for diagnosis, the reported number of diagnostic factors is felt to correlate well with the likelihood of bacterial infection [26].

A Canadian Medical Association evidence-based review recommended a score based on Williams' 5 independent predictor symptoms [27]. Fewer than 2 symptoms ruled out ABRS (positive predictive value [PPV], < 40%), 4 or more symptoms ruled in ABRS (PPV, 81%), and 2 or 3 symptoms (PPV, 40%-63%) suggested that radiography might be beneficial to clarify the diagnosis.

More recent studies have emphasized limitations of clinical findings alone and have either introduced new diagnostic elements or else assessed the accuracy of existing symptoms. In a study of 50 patients with upper respiratory tract symptoms of at least 1 week and self-suspected acute maxillary sinusitis, no distinct clinical signs or symptoms were identified that increased diagnostic accuracy [28]. The sensitivity and specificity of the usual clinical signs and symptoms ranged from 0.04 to 0.74 in a small prospective study that defined acute sinusitis (not necessarily bacterial) as 1 or more sinuses with an air fluid level or complete opacification [29]. A history of facial pain and sinus tenderness on percussion were inversely associated with sinusitis (likelihood ratio [LR] < 1.0). Positive LRs were 1.89 (95% confidence interval [CI], 1.06 to 3.39) for symptom duration longer than 10 days, 1.47 (CI, 0.93 to 2.32) for purulent nasal secretions on history, 2.11 (CI, 1.23 to 3.63) for oropharyngeal red streak in the lateral pharyngeal recess, 1.89 (CI, 1.08 to 3.32) for transillumination, and 1.22 (CI, 0.08 to 18.64) for otitis media.

Although transillumination is not considered accurate in the diagnosis of acute rhinosinusitis (ARS),[16] visualization of purulent secretions from the middle meatus using a short wide speculum has been reported to be highly predictive of ARS [25]. Young et al suggested that purulent nasal discharge, signs of pus in the nasal cavity, or sore throat are better criteria than radiography for selecting patients who would benefit from antibiotic therapy [30].

Taken together, these results emphasize the difficulty of making an accurate diagnosis of sinusitis but support existing consensus that symptoms with duration-based criteria are the best currently available tool.

#### Symptom Severity

**Statement 2**: Determination of symptom severity is useful for the management of acute sinusitis, and can be based upon the intensity and duration and impact on patient's quality of life.

**Strength of evidence**: Option

**Strength of recommendation**: Strong

**Rationale**: Although most of the emphasis of diagnosis has been placed upon differentiating between viral and bacterial causes of sinusitis, or when bacterial sinusitis is diagnosed, little attention has been devoted to determining the severity of symptomatology as measured by its impact on the patient's quality of life. While guidelines for determining severity of sinusitis have not been extensively studied [19], it is clear that a need for this exists. These guidelines recommend determining the severity of sinusitis, whether viral or bacterial, based upon the intensity and duration of symptoms and their impact on the patient's quality of life.

Symptom severity can be generally categorized as:

• Mild: occasional limited episode

• Moderate: steady symptoms but easily tolerated

• Severe: hard to tolerate and may interfere with activity or sleep.

#### Radiological Imaging

**Statement 3**: Radiological imaging is not required for the diagnosis of uncomplicated ABRS. When performed, radiological imaging must always be interpreted in light of clinical findings, as radiographic images cannot differentiate other infections from bacterial infection and changes in radiographic images can occur in viral URTIs.

Criteria for diagnosis of ABRS are presence of an air/fluid level or complete opacification. Mucosal thickening alone is not considered diagnostic. Three-view plain sinus X-rays remain the standard. Computed tomography (CT) scanning is mainly used to assess potential complications or where regular sinus X-rays are no longer available.

Radiology should be considered to confirm a diagnosis of ARBS in patients with multiple recurrent episodes, or to eliminate other causes.

**Strength of evidence**: Moderate

**Strength of recommendation**: Strong

**Rationale**: Studies demonstrate that abnormal images of the sinuses cannot stand alone as diagnostic evidence of bacterial rhinosinusitis. Radiologic changes such as simple mucosal thickening are present in most cases of acute viral infections of the upper respiratory tract when sensitive detection methods such as CT scan are used. Incidental findings of mucosal thickening can also be seen in a high percentage of asymptomatic individuals.

In 1994, Gwaltney et al found that abnormalities of the paranasal sinuses on CT scan are extremely common in young adults with acute uncomplicated viral URTIs [14]. Another study reported that abnormalities on CT scans were common even among the general population [31]. Furthermore, radiographic findings of inflammation demonstrating chronic rhinosinusitis (CRS) are found in 27% to 42% of asymptomatic individuals [32,33]. Taken together, these studies highlight the need to correlate clinical presentation with radiographic results when imaging is used to diagnose ABRS.

**Statement 4**: Urgent consultation should be obtained for acute sinusitis with unusually severe symptoms *or *systemic toxicity *or *where orbital or intracranial involvement is suspected.

**Strength of evidence**: Option

**Strength of recommendation**: Strong

**Rationale**: Extension of disease beyond the confines of the sinuses is a medical emergency and requires aggressive assessment, medical therapy, and potential surgical drainage. Individuals with suspected complications should be urgently referred to a setting with appropriate imaging facilities and qualified specialty care.

Red flags for urgent referral include:

• Systemic toxicity

• Altered mental status

• Severe headache

• Swelling of the orbit or change in visual acuity.

Orbital and intracranial complications are the most feared complications of both acute and chronic rhinosinusitis. In the pre-antibiotic era, 20% of patients with orbital cellulitis went blind and 17% of patients died from intracranial sepsis [34]. Even in the current era, complications can result in permanent blindness or death if not treated appropriately and aggressively. Visual loss from sinusitis was reported at a rate of up to 10% in a 1991 study [35].

Periorbital or orbital cellulitis is the most common complication of ABRS and most often caused by acute ethmoid and/or frontal disease [36,37]. Infection spreads from the sinuses to the orbit with relative ease [38,39]. Periorbital cellulitis is seen on CT as soft tissue swelling and manifests as orbital pain, edema, and high fever. If not aggressively treated, it may spread beyond the orbital septum. Postseptal inflammation involves structures of the orbit with the development of proptosis, limitation of ocular motion, pain and tenderness, and conjunctival chemosis. A subperiosteal or orbital abscess may result in ophthalmoplegia (globe becomes fixed as a result of extra-ocular muscle paralysis) and diminished visual acuity. A CT scan showing evidence of an abscess, or lack of clinical improvement after 24 to 48 hrs of intravenous antibiotics are indications for surgical exploration and drainage. Blindness may result from central retinal artery occlusion, optic neuritis, corneal ulceration, or pan-ophthalmitis.

Altered mental status and non-specific signs characterized by high fever, frontal or retro-orbital migraine, and the presence of generic signs of meningeal irritation warrant immediate consultation with an Ear Nose Throat (ENT) specialist and CT scanning (with contrast). Infection can spread from the sinuses to the intracranial structures [40]. Intracranial complications can include osteomyelitis of the frontal bone (Pott's puffy tumor), meningitis, subdural empyema, epidural abscess, brain abscess, and cavernous sinus thrombosis. The mortality rate for intracranial complications ranges from 20% to 60% [41]. High-dose, long-term intravenous antibiotic therapy followed by endoscopic drainage or craniotomy and surgical drainage are usually required for successful treatment [42].

Because of the serious nature of complications, patients with suspected complications of ABRS should be immediately referred to an otolaryngologist with appropriate consultation from other services, including (but not limited to) ophthalmology, neurosurgery, and infectious diseases.

#### Microbiology of ABRS

**Statement 5**: Routine nasal culture is not recommended for the diagnosis of ABRS. When culture is required for unusual evolution, or when complication requires it, sampling must be performed either by maxillary tap or endoscopically-guided culture.

**Strength of evidence**: Moderate

**Strength of recommendation**: Strong

**Rationale**: Sinus puncture and aspiration remain the gold standard for determining the etiology of ABRS. However because of the invasive nature of sinus puncture required for bacterial studies, this procedure is rarely performed.

The bacterial etiology of ABRS has been well defined by numerous studies dating back almost 50 years. Typically, the findings between investigators have been concordant [5,43-46]:

• Sinus puncture and aspiration remain the gold standard for determining the etiology of ABRS, but are rarely performed due to the invasive nature of sinus puncture

• Cultures obtained from the nasal passages do not provide any diagnostic value

• ABRS can be differentiated from viral etiology by a sinus aspirate that shows the presence of >10^4 ^colony forming units of bacteria/mL or if polymorph nuclear cells in sinus fluid exceeds 5000 cells/mL

• Lower quantities of bacteria may represent early stages of infection.

Comparisons of endoscopically-directed middle meatus cultures (EDMM), a less invasive approach to bacterial sampling, with maxillary sinus aspirate (MSA; the gold standard) have reported similar results [47-49]. A meta-analysis comparing the sensitivity and specificity of EDMM with MSA for ABRS reported that EDMM had a sensitivity of 81%, specificity of 91%, and overall accuracy of 87% compared with MSA [50]. Study authors concluded that EDMM was a reliable alterative to MSA for obtaining cultures from patients with suspected ABRS.

#### Take Home Points

ABRS is a bacterial infection of the paranasal sinuses characterized by:

• Sudden onset of symptomatic sinus infection

• Symptom duration > 7 days

• Length of episode < 4 weeks.

Major symptoms (**PODS**):

• Facial **P**ain/pressure/fullness

• Nasal **O**bstruction

• Nasal purulence/discolored postnasal **D**ischarge

• Hyposmia/anosmia (**S**mell).

Diagnosis requires the presence of ≥ 2 PODS, one of which must be O or D, and symptom duration of > 7 days without improvement.

Diagnosis is based on history and physical examination:

• Sinus aspirates or routine nasal culture are not recommended

• Radiological imaging is not required for uncomplicated ABRS.

The severity of sinusitis, whether viral or bacterial, should be based upon the intensity and duration of symptoms and their impact on the patient's quality of life.

Because complications of ABRS can elicit a medical emergency, individuals with suspected complications should be urgently referred for specialist care.

Red flags for urgent referral include:

• Systemic toxicity

• Altered mental status

• Severe headache

• Swelling of the orbit or change in visual acuity.

### Bacteriology

**Statement 6**: The 2 main causative infectious bacteria implicated in ABRS are *Streptococcus pneumoniae *and *Haemophilus influenzae.*

**Strength of evidence**: Strong

**Strength of recommendation**: Strong

**Rationale**: The bacteriology of ABRS in adults has been well documented in multiple clinical trials and mainly involves *S pneumoniae *and *H influenzae*, with a small percentage of other agents such as *Moraxella catarhallis *and *Staphylococcus aureus*. The causative role of these less common pathogens has not been well established.

#### *Streptococcus pneumoniae *and *Haemophilus influenzae*

In virtually every study, *S pneumoniae *and *H influenzae *remain the 2 most predominant pathogens cultured from the maxillary sinus, typically accounting for more than 50% of cases [5,43-46]. Between 1975 and 1989, Gwaltney et al demonstrated that the most common pathogens in patients with ABRS were *S pneumoniae *(41%) and *H influenzae *(35%) [44]. Several years later, the same author compiled data from 8 additional studies and again *S pneumoniae *and *H influenzae *remained the most frequent pathogens isolated from diseased maxillary sinuses [5]. More recent data has borne out the results of historical studies [51,52]. Although limited data exist, cultures obtained from other sinus cavities appear to correlate with findings obtained from the maxillary sinus [53]. *H influenzae *and *S pneumoniae *are most often isolated in pure culture but are occasionally found together or in combination with other organisms [45,46,52,54]. *H influenzae *strains isolated from sinus puncture are almost exclusively unencapsulated (non-typeable).

#### Other Pathogens

*M catarrhalis *is infrequently isolated from the adult population, but is more common in children where it accounts for approximately 25% of bacteria [55]. Other organisms commonly isolated include *S pyogenes, S aureus*, gram-negative bacilli, and the oral anaerobes [5,51,52].

An exception appears to be acute sinusitis of odontogenic origin, where anaerobic organisms appear to predominate. In 1 study, anaerobes were recovered in 50% of patients, and predominately consisted of *Peptostreptococcus *spp, *Fusobacterium *spp, and *Prevotella *spp [53]. Mixed anaerobic and facultative anaerobic bacteria were recovered in an additional 40% of patients, including the alpha-haemolytic *Streptococci*, microaerophilic *Streptococci*, and *S aureus*. Only 5% of odontogenic specimens grew either *S pneumoniae *or *H influenzae*. Beta-lactamase producing bacteria were isolated from 10 of 20 specimens.

#### Severity of Disease Linked to Pathogen

Several recent studies have increased our understanding of the bacterial etiology associated with ABRS. At least 1 study has demonstrated that severity of disease is dependent on the infecting pathogen [56]. Compared with patients infected with *H influenzae*, patients infected with *S pneumoniae *showed a significantly higher incidence of severe disease (39.2% vs 23.6%, *P *= .0097) and complete sinus opacification (46.2% vs 29.2%, *P *= .0085). Another study has suggested that although *S pneumoniae *and *H influenzae *remain the predominant pathogens, the relative frequency between them may have been altered in adults by the use of the 7-valent pneumococcal vaccine in children [57]. In the 4 years prior to the introduction of the vaccine, isolates obtained from the maxillary sinus of 156 adults predominately grew *S pneumoniae *(46%), followed by *H influenzae *(36%). After introduction of the vaccine, the most predominant organisms recovered from 229 adults were *H influenzae *(43%) and then *S pneumoniae *(35%). The difference noted in the rate of recovery of *H influenzae *and *S pneumoniae *between the 2 time frames was statistically significant (*P *< .05).

#### The Rise of Resistant Bacteria

**Figure 2:**
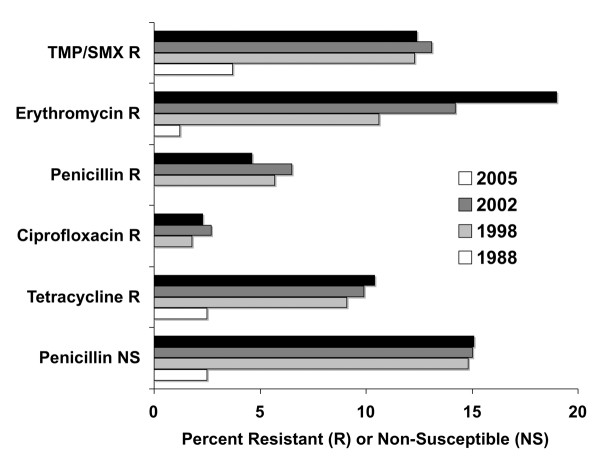
**Trends in Antimicrobial Resistance in Canada **[58,59]

Recent reviews of antimicrobial resistance trends highlight the increasing rates of penicillin, macrolide, and multi-drug resistant *S pneumoniae *in community-acquired respiratory tract infections. Ongoing cross-Canada surveillance has reported increased non-susceptibility and resistance since 1988 (Figure 2) [58,59]. In 2007, the prevalence of penicillin non-susceptibility in Canada was approximately 17% [60]. However, amoxicillin remains active against *S pneumoniae*, with the rate of resistance remaining under 2% [57,61]. Also, despite the increasing use of levofloxacin, moxifloxacin and gatifloxacin, resistance to ciprofloxacin has remained stable [58]. It should be noted that resistance to erythromycin implies cross-resistance to the newer macrolides, clarithromycin and azithromycin. Resistance to the newer fluoroquinolones (levofloxacin and moxifloxacin) remains very low (< 2%) [58].

Higher levels of beta-lactamase production in *H influenzae and M catarrhalis *have been reported [62]. Also, since the introduction of the 7-valent pneumococcal vaccine in children, there has been a shift in the causative agent of adult community acute maxillary sinusitis. Specifically, there is a trend of decreased recovery of *S pneumoniae *resistant to penicillin from 41% to 29% and an increase in beta-lactamase producing *H influenzae *from 33% to 39% [57].

The primary concern for *H influenzae *is ampicillin resistance, mediated by the production of a beta-lactamase. Approximately 19% of *H influenzae *produce a beta-lactamase [63]. *H influenzae *remains predictably susceptible to amoxicillin-clavulanate, the cephalosporins, and the fluoroquinolones [63]. Trimethoprim-sulfamethoxazole (TMP/SMX) and clarithromycin resistance reported from Canadian laboratories are approximately 14% and 2%, respectively. Higher levels of beta-lactamase production in *H influenzae and M catarrhalis *have been reported [62].

Almost 95% of *M catarrhalis *produce a beta-lactamase resulting in penicillin resistance. Aside from the amino-penicillins, *M catarrhalis *remains predictably susceptible to virtually all other antibiotics.

Methicillin-resistant *Staphylococcus aureus *(MRSA) is typically considered a multi-drug resistant pathogen. MRSA had a 2.7% incidence in a study from Taiwan, with nasal surgery being the most important risk factor in adults and prior antibiotic use as the major risk factor in children [64]. Community acquired MRSA (CA-MRSA) strains are resistant to all beta-lactam agents, but typically remain susceptible to TMP/SMX, doxycycline, and clindamycin [65]. At least 1 study has demonstrated that 4% of ABRS infections were associated with CA-MRSA in the United States [66].

Clinicians should be cognizant of their local patterns of resistance, as regional variations exist and some provinces report significantly higher rates of resistance than others.

### Treatment of ABRS

#### Role of Antibiotics

**Statement 7**: Antibiotics may be prescribed for ABRS to improve rates of resolution at 14 days and should be considered where either quality of life or productivity present as issues, or in individuals with severe sinusitis or comorbidities. In individuals with mild or moderate symptoms of ABRS, if quality of life is not an issue and neither severity criterion nor comorbidities exist, antibiotic therapy can be withheld.

**Strength of evidence**: Moderate

**Strength of recommendation**: Moderate

**Rationale**: Antibiotics may speed time to resolution of symptoms in individuals with ABRS. However, overall response rates evaluated at 14 days are similar for both antibiotic-treated and untreated patients. Incidence of side effects, mainly digestive, increases with antibiotic administration.

The goals of treatment for ABRS are to relieve symptoms by controlling infection, decreasing tissue edema, and reversing sinus ostial obstruction to allow drainage of pus [67]. Treatment approaches are shown in Figure 1. There is no evidence to support prophylactic antibiotic therapy.

Many studies support the efficacy of antibiotics for acute sinusitis. Results from a meta-analysis of 6 randomized, placebo-controlled trials of amoxicillin or folate inhibitors for acute sinusitis or acute exacerbation of chronic sinusitis reported that antibiotics decreased risk of clinical failure by half (risk ratio [RR] = 0.54; 95% CI, 0.37-0.79) compared with placebo treatment [68]. A 2009 meta-analysis of 6 placebo-controlled studies reported a RR of 0.66 (95% CI, 0.44 to 0.98) for antibiotic use versus placebo, but noted questionable clinical significance of the results as both groups had high cure rates (80% placebo vs 90% antibiotics) [69]. Their conclusions agreed with the previous meta-analysis in that clinical failure was significantly less frequent with antibiotics compared with placebo at 7 to 15 days of follow up (RR, 0.74; CI, 0.65 to 0.84). In a third meta-analysis, 16 randomized, placebo-controlled studies of antibiotics for the treatment of presumed ABRS were included [70]. This study used a random effect model odds ratio (OR) and reported a higher proportion of improvement or cure (OR = 1.60, 95% CI, 1.31 to 1.96), but also a higher rate of adverse events (OR = 1.94, 95% CI, 1.29-2.92) for the antibiotic group versus the placebo group.

Although antimicrobial therapy is recommended for the management of ABRS, this recommendation is not without controversy [15,16,69-71]. In a meta-analysis of studies enrolling patients with suspected ABRS not confirmed by imaging, laboratory testing, or cultures, analysis of individual patient data resulted in an OR of 1.37 (95% CI, 1.13 to 1.66) for antibiotic use versus placebo [72]. The calculated number needed to treat was 15. Study authors concluded that clear justification for antibiotic treatment was lacking when ABRS was based on clinical signs and symptoms. However, because the analysis included studies of patients who had not had X-rays of the sinuses, and studies enrolled patients with obvious viral infection, the meta-analysis missed an opportunity to assess antibiotic efficacy in patients who were clearly likely to benefit from treatment [73]. In another meta-analysis of patients with symptoms of acute sinusitis or rhinitis (10 studies) or acute rhinorrhea (3 studies), symptom duration averaged 8.1 days (studies ranged from a median of 4.5 days to a mean of 15.4 days), and diagnosis was made from signs and symptoms in over half of the studies. Although cure or improvement rates were significantly better for the antibiotic group at 7 to 12 days, there was no difference between treatment groups at 15 days, suggesting that there was no difference between antibiotics and placebo on patient outcomes. However, the meta-analysis included studies of patients who likely had viral rhinosinusits, in which antibiotics would be ineffective, thus reducing the ability to assess drug efficacy on patients most likely to benefit from treatment [74]. A long-term objection to interpretation of placebo versus antibiotic studies of acute sinusitis has been that the presumed effectiveness of antibiotics in the management of bacterial rhinosinusitis is diluted by the large number of individuals with viral disease participating in these trials. However, a recent study has suggested that even in cases of bacterial rhinosinusitis confirmed by sinus aspirate obtained via puncture, antibiotics are no better than placebo. In this study, patients with positive bacterial cultures for ABRS reported that while 5-day moxifloxacin treatment led to numerically fewer clinical failure rates versus placebo (19.2% vs 33.3%, respectively), the difference was not statistically significant (*P *= .122) [75]. Although the findings suggested a trend for faster symptom resolution and lower failure rates for antibiotic-treated individuals, they did not confirm the absolute utility of antibiotic treatment compared with placebo.

Combined, the various studies and meta-analyses do suggest that antibiotic use, in the setting of ABRS, may speed time to symptom resolution, but that little effect is noted upon ultimate outcome, with similar rates of resolution.

#### Take Home Points

Microbiology of ABRS:

• Main causative bacteria are *S pneumoniae *and *H influenzae*

• Minor causative bacteria are *Moraxella catarhallis *and *S aureus*

○ *M catarrhalis *is infrequent in the adult population, but accounts for about 25% of bacteria in children

• Anaerobic organisms appear to predominate in acute sinusitis of odontogenic origin.

Role of antibiotic therapy in individuals with ABRS:

• Goals of treatment are to relieve symptoms by:

○ Controlling infection

○ Decreasing tissue edema

○ Reversing sinus ostial obstruction to allow drainage of pus

• Antibiotics may be prescribed to improve rates of symptom resolution

○ Overall response rates are similar for antibiotic-treated and untreated individuals.

• Antibiotics should be considered for individuals:

○ With severe sinusitis or comorbidities

○ Where quality of life or productivity are issues

• Incidence of side effects, mainly digestive, increases with antibiotic administration.

#### Choosing an Antibiotic

**Statement 8**: When antibiotic therapy is selected, amoxicillin is the first-line recommendation in treatment of ABRS. In beta-lactam allergic patients, trimethoprim-sulfamethoxazole (TMP/SMX) combinations or a macrolide antibiotic may be substituted.

**Strength of evidence**: Option

**Strength of recommendation**: Strong

**Statement 9**: Second-line therapy using amoxicillin/clavulanic acid combinations or quinolones with enhanced gram positive activity should be used in patients where risk of bacterial resistance is high, or where consequences of failure of therapy are greatest, as well as in those not responding to first-line therapy. A careful history to assess likelihood of resistance should be obtained, and should include exposure to antibiotics in the prior 3 months, exposure to daycare, and chronic symptoms.

**Strength of evidence**: Option

**Strength of recommendation**: Strong

**Rationale**: A comprehensive knowledge of the common etiologies associated with ABRS and the prevalence of antibiotic resistance among these pathogens is paramount to select appropriate treatment. Because antibiotic selection will almost always be made in the absence of bacterial cultures to guide management, activity against the suspected pathogen should be considered.

Some important considerations for choosing an antibiotic include: the suspected or confirmed etiology, medical history, Canadian patterns of antimicrobial resistance, tolerability, convenience, and cost of treatment. It should also be noted that an individual's medical history is an important factor in treatment strategy. Patients who are at increased risk of bacterial resistance and complications due to underlying disease (eg, diabetes, chronic renal failure, immune deficiency) should not be treated the same as otherwise healthy adults with ABRS. Underlying systemic disorders place patients with ABRS at increased risk of recurrence, antibiotic resistance, and complications.

Studies have reported that expensive antibiotics were no more effective than amoxicillin or folate inhibitors for acute uncomplicated sinusitis in otherwise healthy adults [68]. A meta-analysis of 3338 patients from 16 randomized comparative non-placebo studies concluded that differences between antimicrobial agents are small in otherwise healthy adults and adolescents, and therefore an inexpensive antibiotic should initially be chosen [61]. Current evidence based on randomized controlled trials suggest comparable efficacy amongst the antibiotics that have been approved for ABRS in Canada [15,16,76-85]. These include amoxicillin, amoxicillin/clavulanate, cefuroxime axetil, clindamycin, TMP/SMX, clarithromycin, ciprofloxacin, levofloxacin, and moxifloxacin [86].

Selection between these different options may be difficult. Current recommendations are made on the basis of presumed efficacy, risk of bacterial resistance, presence of complications, or cost of therapy.

First-line therapy is amoxicillin. Surveillance studies demonstrate that resistance rates to amoxicillin by streptococci remain low and a consistent response remains predicted. Higher doses of amoxicillin are suggested in suspected cases of penicillin-resistant *S pneumoniae *[62]. In patients with a questionable history of beta-lactam allergy, skin testing may be appropriate to confirm or deny sensitivity, as restricting use of penicillin and penicillin derivatives may result in disadvantages to the patient (ie, costs, side effects) [87]. First-line use of macrolides should probably be limited to patients allergic to penicillin.

Individuals with no clinical response within 72 hours may be presumed to be unresponsive to therapy. The possibility of bacterial resistance should be suspected, and therapy should be changed to a second-line antibiotic.

Second-line therapy using fluoroquinolones with enhanced gram-positive activity (ie, levofloxacin, moxifloxacin) or amoxicillin-clavulanic acid inhibitors as initial management may be needed when there are concerns of bacterial resistance or risk of complications in cases of failure due to underlying disease.

Some populations have been found to be at greater risk of harboring penicillin- and macrolide-resistant streptococci. Depending on geographic location and environment, *S pneumoniae *may be resistant to macrolides and TMP/SMX in nearly one third of cases [88]. Compared with control subjects, those with exposure to daycare settings had a 3.79 (CI, 0.85 to 7.77) higher odds of having penicillin-resistant infection [89]. It has been demonstrated that individuals with invasive streptococcal infections and antibiotic use within the past 3 months have a higher rate of antibiotic resistance, particularly in those treated with TMP-SMX (OR, 5.97) or the macrolide azithromycin (OR, 2.78) [90]. Individuals with antibiotic use within the past 3 months, chronic symptoms greater than 4 weeks, or parents of children in daycare have a higher risk of harboring penicillin- and macrolide-resistant bacteria and should be treated accordingly.

Second-line therapy used as initial management is also needed in situations where a higher risk of complication is associated with treatment failure because of underlying systemic disease. Bacterial sinusitis of the frontal and sphenoid sinuses pose a higher risk of complication than maxillary and ethmoid sinusitis and require more aggressive management and surveillance, with first-line therapy consisting of a second-line agent [16]. Individuals with underlying immunosuppressive sites or medications, or with chronic medical conditions, are at increased risk of complications if failure of therapy occurs.

**Statement 10**: Bacterial resistance should be considered when selecting therapy.

**Strength of evidence**: Strong

**Strength of recommendation**: Strong

**Rationale**: Bacterial resistance rates to penicillin and macrolide/streptogramin/licosamide families have increased rapidly over the past decade to the extent that penicillin and macrolide resistance is now common. Failure of therapy secondary to resistant organisms has led to poor clinical outcomes in several well-documented instances.

There is increasing evidence for the association between antimicrobial resistance and adverse patient outcomes [91,92]. Clinicians should enquire about recent antibiotic use and choose an alternate class of antibiotic from that used in the past 3 months [93]. Supporting this approach are new data that have shown that therapy within the past 3 months is a risk factor for pneumococcal resistance. The Toronto Bacterial Network evaluated data from patients in 3339 cases of invasive pneumococcal infection, of whom 563 had a history of antibiotic therapy in the preceding 3 months where the identity of the therapy was known [90]. In the study, recent therapy with penicillin, macrolides, trimethoprim-sulfa, and quinolones (but not cephalosporin) was associated with a higher frequency of resistance to that same agent. Other patient subgroups identified as at risk for infection with resistant bacterial strains included the young (< 2 years of age), the elderly (> 65 years of age), and those with severe underlying disease. These findings emphasize the importance of taking a history of recent antibiotic use and choosing an agent that differs from what the patient had recently received.

#### Take Home Points

There are increasing rates of antibiotic resistance:

• Penicillin-, macrolide-, and multi-drug resistant *S pneumoniae *in community-acquired respiratory tract infections

• Be cognizant of local patterns of antibiotic resistance, as regional variations exist.

Medical history influences treatment choice:

• Identify patients at increased risk of bacterial resistance and complications

○ Those with underlying disease (eg, diabetes, chronic renal failure, immune deficiency)

○ Those with underlying systemic disorders.

Considerations for choosing an antibiotic:

• Suspected or confirmed etiology

• Medical history

• Presence of complications

• Canadian patterns of antimicrobial resistance

• Risk of bacterial resistance

• Tolerability

• Convenience

• Cost of treatment.

Antibiotic choice:

• First-line: amoxicillin

○ In beta-lactam allergy: TMP/SMX or macrolide

• Second-line: amoxicillin/clavulanic acid combination, or quinolones with enhanced gram-positive activity (ie, levofloxacin, moxifloxacin)

○ For use where first-line therapy failed (defined as no clinical response within 72 hours), risk of bacterial resistance is high, or where consequences of therapy failure are greatest (ie, because of underlying systemic disease).

For uncomplicated ABRS in otherwise healthy adults, antibiotics show comparable efficacy.

**Statement 11**: When antibiotics are prescribed, duration of treatment should be 5 to 10 days as recommended by product monographs. Ultra-short treatment durations are not currently recommended by this group.

**Strength of evidence**: Strong

**Strength of recommendation**: Moderate

**Rationale**: Some data support efficacy of shorter durations of therapy; however, none of these short durations have been approved in Canada, and are thus not recommended by this group.

Traditional approaches to antimicrobial management of ABRS focus on courses of therapy of at least 10 days duration [94]. The rationale for this length of therapy originated from studies in tonsillopharyngitis. Potential benefits of short-course therapy include improved compliance, fewer adverse events, reduced risk of treatment failure and bacterial resistance, and reduced cost. A number of studies have investigated short-course therapy with various antibiotics and have demonstrated similar benefit as comparators (Table 6). These studies have been performed using a variety of antibiotics, some recommended, some not presently recommended in these guidelines, and several either not or no longer marketed in Canada. Of note is that in the United States, a 1-day course of azithromycin reported comparable efficacy to the comparator [95]. Despite this result, it is the opinion of the group that a recommendation for ultra-short courses of therapy be reserved until further supporting trials are performed.

**Table 6:** Studies Investigating Alternative Therapy Duration, Dose, or Formulation

Agent	Comparator	Success Rate	Side Effects
***Duration***			

Azithromycin [96]	Amoxicillin/clavulanate	88.8% and 84.9%, vs 84.9%	Higher for amox/clav group
500 mg/d	1500/375 mg/d		
3 or 6 days	10 days		

Azithromycin [95] microspheres	Levofloxacin	92.5% vs 92.8%	Comparable
2 g	500 mg/d		
1 day	10 days		

Azithromycin [97] (meta-analysis)	Amoxicillin, roxithromycin, cefaclor, erythromycin, amoxicillin/clavulanate, clarithromycin, penicillin	Comparable	Comparable
3 or 5 days			

Levofloxacin [98]	Levofloxacin	>90% for both groups	Comparable
750 mg/d	500 mg/d		
5 days	10 days		

Gatifloxacin [99]*	Amoxicillin/clavulanate	74% and 80%, vs 72%	Comparable
400 mg/d	1750/250 mg/d		
5 or 10 days	10 days		

Gemifloxacin [100]	Gemifloxacin	83.5% vs 84.2%	Comparable
320 mg/d	320 mg/d		
5 days	7 days		

***Dosing***			

Amoxicillin/clavulanate [101]	Amoxicillin/clavulanate	88% vs 93%	Comparable
500/125 mg	875/125 mg		
Every 8 hours	Every 12 hours		

***Formulation***			

Clarithromycin [102] ER	Amoxicillin/clavulanate	98% vs 97%	Comparable
1000 mg/d	1750/250 mg/d		
14 days	14 days		

*The fluoroquinolone, gatifloxacin was removed from the market following a study demonstrating potentially life-threatening glycemic events [103].

ER, extended release.

It is of the opinion of the group that 10 days of therapy with an antibiotic is sufficient. Evolution of the disease and symptom response remains similar regardless of shorter or longer courses of antibiotics [104]. Thus, absence of complete cure (improvement in symptoms without complete disappearance of symptoms) at the end of therapy should be expected and should not cause an immediate prescription of a second antibiotic.

#### Alternatives to Antibiotics: Intranasal Corticosteroids (INCS) as Monotherapy

**Statement 12**: Topical INCS can be useful as sole therapy of mild-to-moderate ARS.

**Strength of evidence**: Moderate

**Strength of recommendation**: Strong

**Rationale**: Topical INCS offer an approach to hasten resolution of sinus episodes and clearance of infectious organisms by promoting drainage and reducing mucosal swelling [105]. They are also used to decrease the frequency and severity of recurrent episodes [106]. Concerns regarding safety of treatment with INCS have not been borne out as their use has not been associated with an increased incidence of complications as judged by adverse events or increased rates of infection [105].

Two studies have identified a positive effect from the use of an INCS as sole treatment modality on resolution of ARS. A study of 981 patients with acute uncomplicated RS randomized patients to receive mometasone furoate nasal spray 200 mcg once daily or twice daily for 15 days, amoxicillin 500 mg 3 times daily for 10 days, or placebo [107]. At 14 days, mometasone furoate twice daily significantly improved symptom scores compared with placebo (*P *< .001) and amoxicillin (*P *= .002). Symptom scores were significantly improved beginning on day 2 with mometasone furoate twice daily compared with amoxicillin and placebo. Global response to treatment at day 15 was also significantly improved with mometasone furoate twice daily compared with amoxicillin and placebo. Although treatment failure was lower with mometasone furoate twice daily than with amoxicillin, the difference did not reach statistical significance. In a study assessing quality of life with the SinoNasal Outcome Test (SNOT)-20 questionnaire, 340 patients with acute uncomplicated RS were randomized to mometasone furoate 200 mcg once daily or twice daily, amoxicillin 500 mg 3 times daily, or placebo [108]. After 15 days of treatment, the mometasone furoate 200 mcg twice daily group had significantly improved scores on the SNOT-20 questionnaire compared with the placebo group.

In another study, patients who presented with at least 2 of the Berg criteria were recruited from primary care practices and randomized to 1 of 4 treatment arms: antibiotic plus budesonide, antibiotic plus placebo budesonide, placebo antibiotic plus budesonide, or placebo antibiotic plus placebo budesonide [109]. Interventions were amoxicillin 500 mg thrice daily for 7 days and 200 mg of budesonide per nostril once daily for 10 days. Results showed no significant difference between treatment arms (OR = 0.99, 95% CI, 0.57 to 1.73 for antibiotic vs placebo; OR = 0.93, 95% CI, 0.54 to 1.62 for budesonide vs placebo). Authors concluded there was no place for these agents in the treatment of ARS in primary care. However, because the median days of symptom duration at presentation was shorter (7 days, with a range of 4 to 14 days) than currently recommended, the patient population may have included a greater proportion than usual of viral rather than bacterial sinusitis [110], thus limiting the ability to detect the benefit of treatments on bacterial episodes.

Although there is limited evidence for and against the use of INCS as monotherapy in the treatment of ABRS, it remains an interesting treatment approach. INCS currently offers a novel option that may be explored based on limited evidence suggesting benefit. In the context of conflicting results between different trials, the use of INCS with established dosing requirements indicated for ABRS may be preferable. Additional clinical trials and further experience in coming years will better discern its role in the management of ABRS.

#### Management of Failures of First-Line Therapy

**Statement 13**: Treatment failure should be considered when patients fail to respond to initial therapy within 72 hours of administration. If failure occurs following use of INCS as monotherapy, antibacterial therapy should be administered. If failure occurs following antibiotic administration, it may be due to lack of sensitivity to, or bacterial resistance to, the antibiotic, and the antibiotic class should be changed.

**Strength of evidence**: Option

**Strength of recommendation**: Strong

**Rationale**: In patients managed with a topical corticosteroid as sole therapy, persistent bacterial infection may be presumed and an antibiotic should be instituted, according to guidelines for selection of an antibiotic. Bacteriologic response to antibiotics should be expected within 48 hours, thus symptoms should at least partially attenuate by 72 hours. If symptoms persist unchanged at this time, failure of response to antibiotic therapy must be considered along with possible resistance [71]. Antibiotic therapy must be adjusted by switching to a second-line antibiotic such as moxifloxacin or amoxicillin/clavulanic acid combination or, in the case of a second-line failure, to another antibiotic class.

Studies using in-dwelling catheters for serial sampling of sinus fluid have reported the time course of antibiotics to eradicate pathogens as ranging from 24 to 72 hours [111-113]. In the absence of at least a partial clinical response by 72 hours, bacterial resistance should be suspected as one of the causes of failure and appropriate measures should be instituted.

#### Take Home Points

Factors suggesting greater risk of penicillin- and macrolide-resistant streptococci:

• Antibiotic use within the past 3 months

○ Choose an alternate class of antibiotic from that used in the past 3 months

• Chronic symptoms greater than 4 weeks

• Parents of children in daycare.

When antibiotics are prescribed, treatment duration should be 5 to 10 days as recommended by product monographs.

• Improvement in symptoms without complete disappearance of symptoms at the end of therapy should be expected and should not cause an immediate prescription of a second antibiotic.

Topical INCS can be useful as sole therapy of mild-to-moderate ARS.

Treatment of first-line therapy failure:

• If symptoms do not at least partially attenuate by 72 hours after INCS monotherapy:

○ Administer antibiotic therapy

• If symptoms do not at least partially attenuate by 72 hours after antibiotic administration:

○ Bacterial resistance should be considered, *and*

○ Antibiotic class should be changed

○ Switch to a second-line antibiotic, such as

▪ Moxifloxacin

▪ Amoxicillin/clavulanic acid combination

○ In the case of a second-line failure, switch to another antibiotic class.

#### Adjunct Therapy

**Statement 14**: Adjunct therapy should be prescribed in individuals with ABRS.

**Strength of evidence**: Option

**Strength of recommendation**: Strong

**Statement 15**. Topical intranasal corticosteroids (INCS) may help improve resolution rates and improve symptoms when prescribed with an antibiotic.

**Strength of evidence**: Moderate

**Strength of recommendation**: Strong

**Statement 16**. Analgesics (acetaminophen or non-steriodal anti-inflammatory agents) may provide symptom relief.

**Strength of evidence**: Moderate

**Strength of recommendation**: Strong

**Statement 17**. Oral decongestants may provide symptom relief.

**Strength of evidence**: Option

**Strength of recommendation**: Moderate

**Statement 18**. Topical decongestants may provide symptom relief.

**Strength of evidence**: Option

**Strength of recommendation**: Moderate

**Statement 19**. Saline irrigation may provide symptom relief.

**Strength of evidence**: Option

**Strength of recommendation**: Strong

**Rationale**: Analgesics, oral and topical decongestants, topical INCS, and saline sprays or rinses can all help relieve symptoms of both viral and bacterial infections of the upper respiratory passages and can all be suggested for symptomatic relief.

##### Ancillary and Alternative Therapies

Recent reviews suggest that the evidence for use of ancillary therapies is relatively weak, as few prospective randomized clinical trials have been performed to assess their effectiveness. This does not necessarily mean that the therapies are of no benefit, as these have long been a part of clinical practice and may offer benefits. However, the lack of good quality trials supporting their use requires the incorporation of weaker levels of evidence, thus recommendations are derived from extension from first principles and expert opinion.

Based on its effects on inflammation, topical INCS in conjunction with antibiotic therapy have been assessed for their effectiveness in improving resolution of signs and symptoms of rhinosinusitis. In the Cochrane review on this topic, 3 randomized, placebo-controlled studies of the efficacy of 15- to 21-day courses of mometasone furoate, fluticasone propionate, or budesonide for nasal endoscopy-confirmed ARS found limited but positive evidence for INCS as an adjuvant to antibiotics [105]. The symptoms of cough and nasal discharge were significantly improved (*P *< .05) through the second week of treatment for patients receiving budesonide (50 mcg) plus amoxicillin-clavulanate potassium compared with those receiving placebo plus the antibiotic [114]. In patients receiving mometasone furoate (200 mcg or 400 mcg twice daily) plus amoxicillin/clavulanate potassium, total symptom score days 1 to 15 averaged and over the 21-day study period were significantly improved (*P *≤ .017) compared with patients receiving placebo plus antibiotic [115]. In a third study, a 21-day course of fluticasone propionate (200 mcg) plus cefuroxime improved the clinical success rate compared with placebo plus antibiotic (93.5% vs 73.9%, *P *= .009) as well as the speed of recovery (6 days vs 9.5 days, *P *= .01) [106]. No significant steroid-related adverse effects or recurrence rates were reported. Topical INCS thus appear to be safe and to afford an additional benefit when antibiotics are used.

Oral decongestants have been shown to improve nasal congestion and can be used until symptoms resolve, provided there are no contraindications to their use. Topical decongestant use is felt to be controversial and should not be used for longer than 72 hours due to the potential for rebound congestion [9].

There are no clinical studies supporting the use of antihistamines in ABRS [71]. Although 1 randomized controlled trial of human immunodeficiency virus-infected patients with acute or chronic sinusitis reported benefit with the mucolytic agent guaifenesin [116], no benefit was reported in a randomized controlled trial in healthy subjects [117].

There is limited evidence suggesting benefit of saline irrigation in patients with acute sinusitis. Many studies support the role of buffered hypertonic and buffered normal nasal saline to promote mucociliary clearance. In a study of patients with ABRS, thrice-daily irrigation with 3% nasal saline improved mucociliary clearance beginning in week 1 [118]. Moreover, subjects using once daily hypertonic saline nasal irrigation reported significantly improved symptoms, quality of life, and decreased medication use compared with control subjects [119]. However, the impact of saline sprays on nasal airway patency is less clear, with studies variously reporting no impact of saline sprays [120] and improved patency with buffered physiological saline spray [121]. Their impact on symptom improvement is also uncertain, with a study of hypertonic saline spray reporting no improvement in nasal symptoms or illness duration [122]. Saline therapy, either as a spray or high-volume irrigation, has seen widespread use as adjunct treatment despite a limited evidence base. Although the utility of saline sprays remains unclear, the use of saline irrigation as ancillary therapy is based on evidence of modest symptomatic benefit and good tolerability.

##### Complementary and Alternative Medicine

Recent reviews have found limited evidence for alternative and complementary medicine for ABRS [71,123]. Some of these therapies include home-based foods such as soups, fruit juices, teas, nutritional supplements, and herbal remedies. Alternative practices that have failed to show efficacy under scientific trial conditions include acupuncture, chiropracty, naturopathy, aromatherapy, massage, and therapeutic touch. Vitamin C preparations and zinc lozenges are also felt to be controversial [71,123]. Studies of zinc lozenges for the common cold have produced mixed results. A recent meta-analysis of Echinacea preparations has shown some positive effects in reducing duration of respiratory tract symptoms [124].

A recent systematic review comparing placebo with the herbal medications Sinupret or Bromelain as adjunct therapy reported limited evidence of improved symptoms. Further, single randomized controlled trials on Esbetritox, Mytrol, Cineole, and Bi Yuan Shu showed some initial positive evidence [125]. A prospective randomized controlled trial compared the homeopathic medication Sinfrontal with placebo among 56 cases and controls with radiograph-confirmed acute maxillary sinusitis [126]. Participants were allowed saline inhalations, paracetemol, and over-the-counter medications; however antibiotics or other conventional therapies for sinusitis were not permitted. From day 0 to 7, Sinfrontal was associated with greater reduction in sinusitis severity scores compared with placebo (*P *< .001). On day 21, 68.4% of the Sinfrontal group had complete resolution of symptoms versus 8.9% of the placebo group. No recurrence was reported by the end of an 8-week post-treatment observation period. Eight mild-to-moderate adverse events were reported in the Sinfrontal group. Although this data is of interest, further confirmatory studies on the efficacy and safety of herbal medicines are needed before they can be recommended. Physicians must inquire about the use of complementary therapies with their patients due to potential drug interactions with conventional treatments and potential toxicities related to the alternative/complementary therapies themselves.

#### Management of Persistent Symptoms or Recurrent Acute Uncomplicated Sinusitis

**Statement 20**: For those not responding to a second course of therapy, chronicity should be considered and the patient referred to a specialist. If waiting time for specialty referral or CT exceeds 6 weeks, CT should be ordered and empiric therapy for CRS administered. Repeated bouts of acute uncomplicated sinusitis clearing between episodes require only investigation and referral, with a possible trial of INCS. Persistent symptoms of greater than mild-to-moderate symptom severity should prompt urgent referral.

**Strength of evidence**: Option

**Strength of recommendation**: Moderate

**Rationale**: Recurrent ABRS is defined as repeated symptomatic episodes of acute sinusitis (≥4 episodes per year) with clear symptom-free periods in between that correspond to complete resolution between infections. Individuals failing to respond to therapy or recurring with symptoms early following therapy should be judged to have CRS. CRS is an inflammatory disease with symptoms that persist for 8 to 12 weeks. Referral to a specialist is necessary to document CRS with endoscopy or CT. Indications for referral include:

• Persistent symptoms of ABRS despite appropriate therapy, or severe ABRS

• Treatment failure after extended course of antibiotics

• Frequent recurrence (≥4 per year)

• Immunocompromised host

• Evaluation for immunotherapy of allergic rhinitis

• Anatomic defects causing obstruction

• Nosocomial infections

• Biopsy to rule out fungal infections, granulomatous disease, neoplasms.

Furthermore, possible contributing factors (eg, underlying allergy, immunologic propensity for sino-pulmonary infections) must be evaluated.

If confirmation of diagnosis by CT or specialty referral to an ENT specialist for endoscopy is available within 6 weeks, administration of additional therapy may await confirmation of the diagnosis. However, if CT or specialty referral is unavailable within this timeframe, an initial course of therapy for CRS should be given during the wait for investigation and/or referral.

### Prevention

**Statement 21**: By reducing transmission of respiratory viruses, hand washing can reduce the incidence of viral and bacterial sinusitis. Vaccines and prophylactic antibiotic therapy are of no benefit.

**Strength of evidence**: Moderate

**Strength of recommendation**: Strong

**Rationale**: Any strategy that reduces the risk of acute viral infection, the most common antecedent to ABRS, is considered a prevention strategy for ABRS. Because ABRS follows an initial viral rhinitis/sinusitis, reductions in the number of these episodes will help reduce the incidence of bacterial sinusitis. Hand washing has been shown to be effective in reducing person-to-person viral transmission [127]. Patients with recurrent episodes may benefit more from this strategy. In addition to hand washing, educating patients about common predisposing factors may be considered a preventative strategy.

Although vaccines for influenza have an invaluable role in reducing the occurrence and transmission of influenza, no such vaccine exists for the viruses responsible for URTIs. There is no evidence that influenza or pneumococcus vaccination reduces the risk of ABRS [9], which likely reflects the variety of causative pathogens associated with ABRS. Indeed, introduction of the 7-valent pneumococcal vaccine in children led to a shift in the causative pathogens among cases of adult acute sinusitis [57]. However, individuals who meet current guideline criteria for vaccinations are recommended to keep up to date with their vaccines. Prophylactic antibiotics are also not effective in preventing viral episodes or development of subsequent bacterial sinusitis, and are not recommended as routine practice.

#### Immune Testing

**Statement 22**: Allergy testing or in-depth assessment of immune function is not required for isolated episodes but may be of benefit in identifying contributing factors in individuals with recurrent episodes or chronic symptoms of rhinosinusitis.

**Strength of evidence**: Moderate

**Strength of recommendation**: Strong

**Rationale**: Recurrent episodes of ABRS may have underlying contributing factors, including allergic rhinitis and immune deficiencies. In 1 study, patients with CRS or frequent episodes of ARS had a 57% prevalence of positive skin allergy tests [128]. Another study showed that 84% of patients who had surgery for CRS had a positive allergy test, and 58% had multiple allergen sensitivities [129]. Such patients may have increased susceptibility to inflammation of the nose and paranasal sinuses [130]. However, in the treatment of ABRS in primary care, allergy testing is not required for investigating or resolving acute episodes.

#### Take Home Points

Adjunct therapy may provide symptom relief and should be prescribed in individuals with ABRS:

• Topical intranasal corticosteroids (INCS)

• Analgesics (acetaminophen or non-steriodal anti-inflammatory agents)

• Oral decongestants

• Topical decongestants

• Saline irrigation.

The goal of prevention strategies is to reduce the of risk acute viral infection, the most common antecedent to ABRS.

• Techniques:

○ Handwashing

○ Educating patients on common predisposing factors.

For patients with recurrent episodes of ABRS, consider underlying contributing factors:

• Allergy testing to detect allergic rhinitis

• In-depth assessment of immune function to detect immune deficiencies.

## Chronic Rhinosinusitis (CRS)

Adult CRS prompts an estimated 18 to 22 million annual office visits and 545 000 annual emergency room visits in the United States [131]. In a survey study, patients with CRS reported more bodily pain and worse social functioning than patients with other chronic conditions such as chronic obstructive pulmonary disease, congestive heart failure, and back pain [132]. The impact of CRS on patient quality of life is comparable in severity to that of other chronic conditions. As with other chronic diseases, CRS should be proactively managed.

### Definition and Diagnosis

**Statement 23**: CRS is diagnosed on clinical grounds but must be confirmed with at least 1 objective finding on endoscopy or computed tomography (CT) scan.

**Strength of evidence**: Weak

**Strength of recommendation**: Strong

**Rationale**: Symptoms of CRS alone are not sufficient to diagnose CRS because they can be nonspecific and mimicked by several disease entities (eg, upper respiratory tract infection [URTI], migraine). Confirmation of sinus disease using an objective measure is required. Conversely, in the absence of symptoms, diagnosis of CRS based on radiology alone is not appropriate because of a high incidence of radiological anomalies on CT scans in normal individuals. Thus, the presence of symptoms plus an objective finding are necessary.

**Figure 3:**
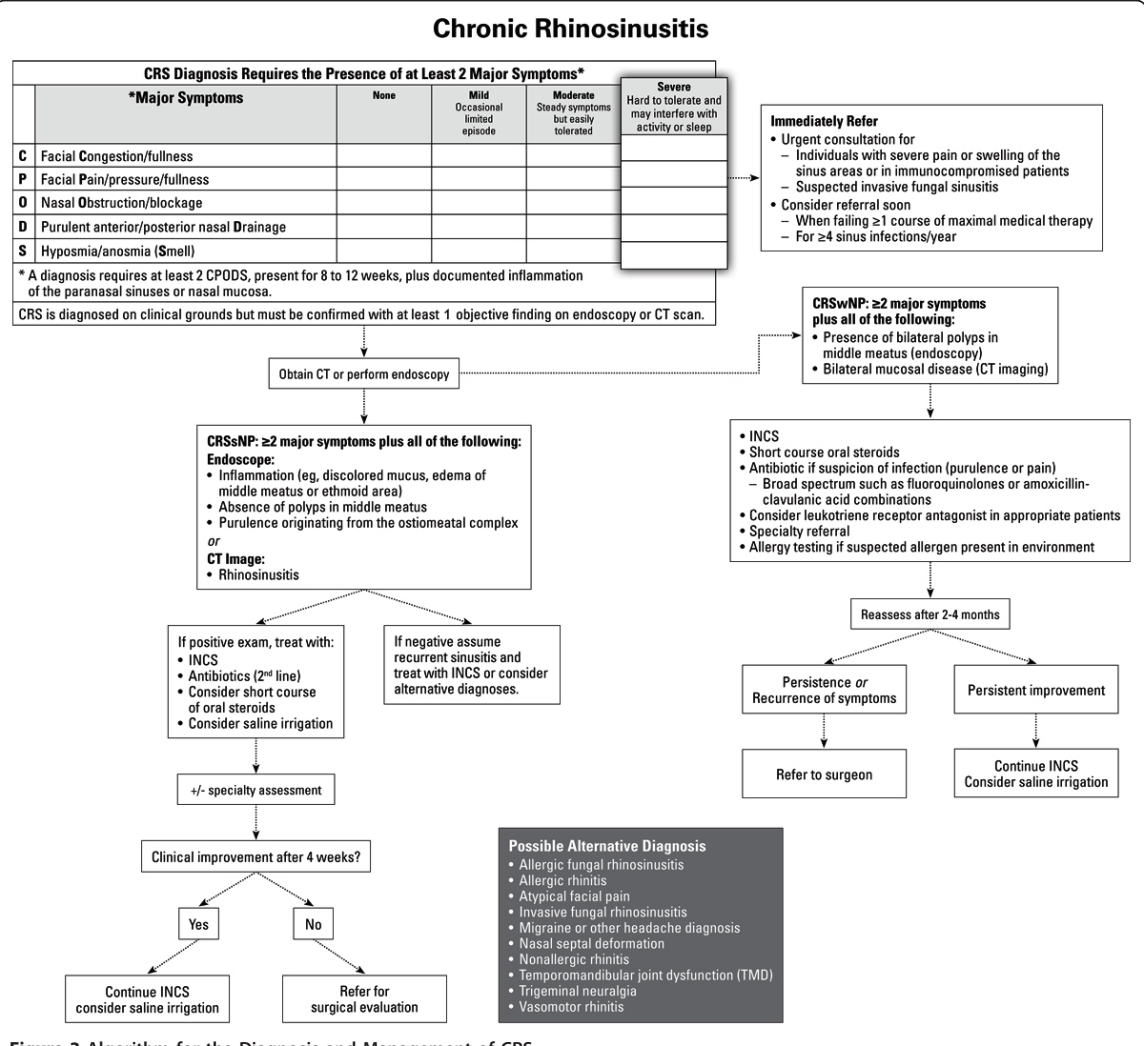
**Algorithm for the Diagnosis and Management of CRS**.

CRS may be defined as an inflammatory disease involving the nasal mucosa and paranasal sinuses [133]. CRS is a symptom-based diagnosis supported by objective documentation of disease by physical findings and diagnostic imaging or sinonasal endoscopy [9,18,133-137]. An algorithm for the diagnosis and management of CRS is presented in Figure 3.

#### Take Home Points

Impact of CRS on patients:

• Significant bodily pain and impaired social functioning

• Quality of life is comparable in severity to that of other chronic conditions

• As a chronic condition, CRS should be proactively managed.

CRS is an inflammatory disease involving the nasal mucosa and paranasal sinuses.

• Symptoms are usually of lesser intensity than those of ABRS

• Symptoms present for 8-12 weeks.

#### Symptoms of CRS

Symptoms of CRS are usually of lesser intensity than those of acute bacterial rhinosinusitis (ABRS) but their duration exceeds the 4 weeks commonly used as the upper limit for the diagnosis of ABRS. A diagnosis of CRS is probable if 2 or more major symptoms are present for at least 8 to 12 weeks along with documented inflammation of the paranasal sinuses or nasal mucosa (Table 7) [9,21,138].

**Table 7:** CRS Diagnosis Requires the Presence of at Least 2 Major Symptoms*

	Major Symptom
**C**	Facial **C**ongestion/fullness

**P**	Facial **P**ain/pressure/fullness

**O**	Nasal **O**bstruction/blockage

**D**	Purulent anterior/posterior nasal **D**rainage (discharge may be nonpurulent, nondiscolored)

**S**	Hyposmia/anosmia (**S**mell) [9,21,136].

*A diagnosis requires at least 2 CPODS, present for 8 to 12 weeks, plus evidence of inflammation of the paranasal sinusus or nasal mucosa.

CRS is diagnosed on clinical grounds but must be confirmed with at least 1 objective finding on endoscopy or CT scan.

CRS can be further categorized based on the absence or presence of nasal polyps (CRS without nasal polyps, CRSsNP; or CRS with nasal polyps, CRSwNP) [137]. Although both are characterized by mucopurulent drainage and nasal obstruction, CRSsNP is frequently associated with facial pain/pressure/fullness whereas CRSwNP is frequently characterized by hyposmia.

A diagnosis of CRSsNP requires the presence of the following:

• At least 2 symptoms *and*

• Inflammation (eg, discolored mucus, edema of middle meatus or ethmoid area) documented by endoscopy *and*

• Absence of polyps in the middle meatus (by endoscopy) *and/or*

• Demonstration of purulence originating from the osteomeatal complex on endoscopy *or *rhinosinustis confirmed by CT imaging.

A diagnosis of CRSwNP requires the presence of:

• At least 2 symptoms *and*

• The presence of bilateral polyps in the middle meatus confirmed by endoscopy *and*

• Bilateral mucosal disease confirmed by CT imaging [137].

Further sub-definitions of sinonasal polyposis include subtypes related to the presence of acetylsalicylic acid (ASA) sensitivity or the presence of eosinophilic mucin, with or without documented immunoglobulin E (IgE)-mediated fungal hypersensitivity [21]. Both subtypes are recalcitrant to treatment.

#### Take Home Points

Major symptoms of CRS:

• Facial **C**ongestion/fullness

• Facial **P**ain/pressure/fullness

• Nasal **O**bstruction/blockage

• Purulent anterior/posterior nasal **D**rainage (discharge may be nonpurulent, nondiscolored)

• Hyposmia/anosmia (**S**mell).

Diagnosis of CRS requires all of the following:

• Presence of ≥ 2 major symptoms (CPODS)

• Symptoms (CPODS) present for at least 8 to 12 weeks

• Documented inflammation of the paranasal sinuses or nasal mucosa

○ Endoscopy

○ CT scan, preferably in coronal view.

CRS subtypes:

• CRS without nasal polpys (CRSsNP), frequently characterized by:

○ Mucopurulent drainage

○ Nasal obstruction

○ Facial pain/pressure/fullness

• CRS with nasal polyps (CRSwNP), frequently characterized by:

○ Mucopurulent drainage

○ Nasal obstruction

○ Hyposmia.

A diagnosis of CRSsNP requires the presence of the following:

• At least 2 symptoms *and*

• Inflammation (eg, discolored mucus, edema of middle meatus or ethmoid area) documented by endoscopy *and*

• Absence of polyps in the middle meatus (by endoscopy) *and/or*

• Purulence originating from the osteomeatal complex on endoscopy *or *rhinosinustis confirmed by CT imaging.

A diagnosis of CRSwNP requires the presence of:

• At least 2 symptoms *and*

• The presence of bilateral polyps in the middle meatus confirmed by endoscopy *and*

• Bilateral mucosal disease confirmed by CT imaging.

#### Physical Examination

**Statement 24**: Visual rhinoscopy assessments are useful in discerning clinical signs and symptoms of CRS.

**Strength of evidence**: Moderate

**Strength of recommendation**: Moderate

**Rationale**: Assessment of the individual with chronic nasal symptoms necessarily includes physical examination of the nasal cavity. Physical examination should be performed with equipment affording good illumination. Although a headlight and nasal speculum is optimal, an otoscope affords an adequate view.

A systematic assessment, evaluation or examination of the nasal septum, inferior turbinates, and middle meatal area should be performed. In the nasal septum, drying crusts, ulceration, bleeding ulceration, and perforation should be identified when present. Attention must be devoted to the identification of anatomic obstructions, unusual aspects of the nasal mucosa, hypertrophy of the turbinates, and/or presence of secretions or nasal masses. Significant septal deflections should be noted. Additionally, color of the nasal mucosa and presence of dryness or hypersecretion should be noted. The normal mucosa is pinkish-orange with a slight sheen demonstrating hydration. Presence of an irregular surface, crusts, diffusely hemorrhagic areas, vascular malformations or ectasias, or bleeding in response to minimal trauma, is abnormal and should warrant specialist assessment.

The turbinates should be carefully inspected. Inferior turbinates should be assessed for hypertrophy. The area of the middle turbinate and the middle meatus adjacent to it between the septum and the lateral nasal wall should be visualized and inspected carefully for the presence of secretions or masses such as nasal polyps. This area may be difficult to visualize and visualization of these areas may be improved by performing vasoconstriction of the nose using a decongestant product such as Dristan^® ^or Otrivin^®^.

Visualization is further improved by the use of sinonasal endoscopy. Sinonasal endoscopy allows for improved visualization of the middle meatal area, the posterior portion of the nose, and nasopharynx and allows better identification of nasal polyposis in the early stages. Although an almost integral component of specialist assessment, it requires specialized equipment and appropriate training. Endoscopy can reveal edema or obstruction of the middle meatus [21]. By allowing visualization of the nasal cavity and sinus openings, nasal endoscopy permits identification of posterior septal deviation and polyps and secretions in the posterior nasal cavity, middle meatus, sphenoethmoidal recess, and direct aspiration of secretions for analysis and culture (see below, endoscopically-directed middle meatus [EDMM]) [9].

#### Methods of Bacterial Recovery

**Statement 25**: In the few situations when deemed necessary, bacterial cultures in CRS should be performed either via endoscopic culture of the middle meatus or maxillary tap but not by simple nasal swab.

**Strength of evidence**: Option

**Strength of recommendation**: Strong

**Rationale**: Endoscopic cultures of the middle meatus are less invasive than maxillary tap. To be of value, these cultures require skilled practitioners to perform the endoscopic examination and sampling to avoid contamination from normal nasal flora or from adjacent structures. Simple swab nasal cultures are of little predictive value. Of note is that when purulent secretions are observed, these should be sampled directly, as culture resulting from a thin stream of pus and an adjacent area can differ. Also, since all specimens are potentially contaminated to varying degrees, proper specimen collection, transport, storage, and processing are key.

Involvement of bacterial biofilm can be difficult to detect, and identification of pathogens relies on analyzing tissue samples with either electron or confocal scanning laser microscopy (CSLM), or indirectly by analyzing the DNA signature for biofilm-forming genes [139,140]. These tests remain primarily research tools and are not currently available in the clinical setting.

##### Maxillary Sinus Aspirate (MSA)

MSA is considered the gold standard for bacterial recovery in acute and chronic sinusitis, but there are still issues of concern. Because the procedure is invasive, it is usually performed by specialists rather than in family practice, and even then only rarely. MSA is recommended if there are serious complications, such as orbital infection, intracranial extension of the infection, or in patients with nosocomial sinusitis. MSAs are usually obtained by puncture through the canine fossa or inferior meatus and are subject to contamination by oral or nasal flora during collection. Because pathogens in various sinuses may differ, MSAs are limited in only being representative of the maxillary sinus. Other concerns with MSAs include patient compliance, discomfort, bleeding, secondary infection, and (rarely) injury to the infraorbital nerve or orbit.

##### Nasal and Postnasal Discharge Culture

Most studies have shown poor correlation of nose and throat cultures with MSAs, and these cultures are generally not recommended in patients with acute or chronic sinusitis.

##### *Endoscopically-Directed Middle Meatus Culture (EDMM)*

EDMM requires specialist expertise and equipment. It is safe and usually painless. The area sampled (the middle meatus) contains the ostiomeatal complex, which provides a common drainage pathway for the maxillary, ethmoid, and frontal sinuses. EDMM cultures are therefore representative of frontal, ethmoid, and maxillary sinuses. However, as with MSAs, EDMMs are subject to contamination with resident nasal flora (including anaerobes), which makes their interpretation subject to clinical situation. Also, pathogenic bacteria such as *S. pneumoniae*, *H. influenzae*, and *S. aureus *may be isolated from asymptomatic patients in the carrier state. The significance of this carrier status is uncertain but in the absence of symptoms, treatment is rarely initiated. EDMM swab and MSA have been shown to be equivalent for detection of pathogens and likelihood of contamination [140,141].

##### Fungal Pathogens

If invasive fungal sinusitis is suspected, prompt diagnosis and treatment are essential. Culture must be requested promptly because these infections are life threatening and usually require emergency surgery. However, results of culture are rarely available to assist with decision-making, and diagnosis is most frequently made on the basis of Gram staining and frozen sections demonstrating the characteristic branching hyphae arrangement. Biopsies for Gram stain and culture (aerobic and anaerobic bacterial culture plus fungal culture) and for histopathology and special stains are key.

##### Conclusions for Bacterial Recovery

Although postnasal/nasal discharge is common, routine cultures of such are discouraged and empiric therapy is recommended. If a patient fails multiple courses of empiric therapy, they should be referred to an otolaryngologist for evaluation, which usually includes sinonasal endoscopy. If purulent material is identified, diagnostic culture may be made by EDMM. When situation warrants it, such as for complications or in nosocomial intensive care unit sinusitis, MSA may be performed.

#### Radiological Imaging

**Statement 26**: The preferred means of radiological imaging of the sinuses in CRS is the CT scan, preferably in the coronal view. Imaging should always be interpreted in the context of clinical symptomatology because there is a high false-positive rate.

**Strength of evidence**: Moderate

**Strength of recommendation**: Strong

**Rationale**: Conventional X-ray images do not adequately image the ethmoid sinuses or the osteomeatal complexes, which are key to the development and persistence of CRS, and when clinically indicated, may thus be assessed with CT scanning. However, positive CT findings alone are not indicative of CRS in the absence of signs or symptoms given the high prevalence of mucosal changes accompanying URTIs and/or asymptomatic changes in the non-diseased population.

CT is an important means of providing objective evidence for the diagnosis of CRS but must absolutely be correlated with clinical and endoscopic findings to interpret them meaningfully. Several studies have demonstrated that CT cannot be used as a sole indicator for CRS [31-33]. Further studies have reported either no correlation [142] or a low correlation [143-145] between symptoms and CT findings. These results reflect the need to interpret imaging results in the context of persistent symptoms to provide an accurate diagnosis. Thus CT should not be used as the sole criteria for determining the need for surgical intervention, but rather should be used as an objective tool for confirming the diagnosis of CRS and for surgical planning. CT scans should be ordered after failure of maximal medical management and/or for the planning of surgery. A complete CT scan should be obtained if the physician is contemplating surgical intervention, and the scan ideally should be at a minimum resolution of 3 mm coronal slices, if not more detailed. Reconstruction in the sagittal plane may help with performance of surgery, particularly in the area of the frontal sinus.

#### Take Home Points

Visual assessments include:

• Physical examination of the nasal cavity with equipment affording good illumination

○ Headlight and nasal speculum

○ Otoscope.

Examination of:

• The nasal septum

○ Identify drying crusts, ulceration, bleeding ulceration, and perforation, anatomic obstructions, unusual aspects of the nasal mucosa, and/or presence of secretions or nasal masses

○ Note significant septal deflections, and color of the nasal mucosa and presence of dryness or hypersercretion. The normal mucosa is pinkish-orange with a slight sheen demonstrating hydration

○ Presence of an irregular surface, crusts, diffusely hemorrhagic areas, vascular malformations or ectasias, or bleeding in response to minimal trauma, is abnormal and should warrant specialist assessment

• Inferior turbinates:

○ Assess for hypertrophy

• Middle meatal area:

○ Inspect the area of the middle turbinate and the middle meatus adjacent to it between the septum and the lateral nasal wall for the presence of secretions or masses such as nasal polyps

○ Visualization of these areas may be improved by performing vasoconstriction of the nose using a decongestant product (eg, Dristan^® ^or Otrivin^®^). Sinonasal endoscopy may improve visualization.

### Pathophysiology

**Statement 27**: CRS is an inflammatory disease of unclear origin where bacterial colonization may contribute to pathogenesis. The relative roles of initiating events, environmental factors, and host susceptibility factors are all currently unknown.

**Strength of evidence**: Weak

**Strength of recommendation**: Moderate

**Rationale**: Intense inflammation with eosinophilic, neutrophilic, and lymphocytic infiltrations and upregulation of numerous T helper (Th) cell type 2-associated cytokines has been well documented in biopsy samples of CRS. The disease process has several similarities with asthma, including infiltration of a similar population of inflammatory cells, cytokine profile, and evidence of tissue remodeling. The role of bacteria remains imprecise in the face of frequent negative cultures, however a role for colonization of *S. aureus *contributing to disease via superantigenic stimulation has been proposed. Emerging evidence on bacterial biofilms may explain frequent negative cultures and change our future understanding of the role of bacteria in CRS.

The last 10 years have witnessed new insights into the inflammatory mechanisms of CRS. Investigation of the inflammatory roles of cytokines and chemokines has shed considerable light on the pathogenesis of this disease. It is now widely documented that T lymphocytes and the activated eosinophils are prominent within the sinus mucosa of patients with CRS, especially in atopic patients. Recruitment and activation of the inflammatory cell infiltrate has largely been attributed to the effects of Th2 cytokines (namely interleukin [IL]-4, IL-5, IL-13, and granulocyte monocyte-colony stimulating factor), and the eosinophil-associated chemokines, eotaxin, and monocyte chemotactic proteins.

Evidence that CRS subtypes have distinct pathogenetic mechanisms, and may represent distinct diseases, has been suggested in biomarker studies of nasal secretions. In 1 study, IL-5 and nasal IgE were significantly associated with CRSwNP but not with CRSsNP or acute rhinosinusitis (ARS) [146]. Another study extended these findings, reporting that CRSwNP had Th2 polarization and a higher prevalence of IL-5, IgE, eosinophils, eotaxin, and eosinophil cationic protein, whereas CRSsNP had Th1 polarization and higher levels of interferon-gamma and transforming growth factor (TGF)-beta [147]. These results indicate that cytokine and mediator profiles may be useful in differentiating between disease entities.

Recruitment and persistence of adaptive immune responses resulting in the development of the clinical symptoms characteristic for CRS may reflect dysfunction of the nasal epithelium and its subsequent inability to properly coordinate immune responses to foreign matter [148]. Epithelial cells in patients with CRS were found to have altered expression and function of Toll-like receptors, production of factors involved in control of innate immunity, and in the functional regulation of local adaptive immunity [148-151].

Due to the heterogeneity of the pathogenesis and the clinical presentation of CRS, it has been suggested that CRS be considered a syndrome with persistent characteristic symptoms instead of as a discrete disease entity [148].

#### Allergy and Inflammation

The inflammatory disease of the nasal and paranasal sinus mucosa is classified as allergic and non-allergic, depending on the presence or absence of atopy. The immunopathologic mechanisms underlying the development of CRS in allergic patients are largely related to the effects of Th2 cytokines and their corresponding receptors. In contrast, a combination of Th1 and Th2 cytokines seems to orchestrate the inflammatory response in non-allergic CRS patients. Similar observations have been made in CRS with and without nasal polyposis [152,153]. Despite these distinct mechanisms, the common outcome in CRS, in both atopic and non-atopic patients, is an intense eosinophilic infiltration. Production of IgE, while present in allergic CRS, has also been reported in CRS even in the absence of history of allergy and the presence of a negative skin test [154].

Remodeling, or structural changes, associated with chronic inflammation include epithelial changes, increased deposition of extracellular matrix proteins (eg, collagen), and increased expression of growth factors and profibrotic cytokines (eg, IL-6, IL-11, IL-17, TGF-beta, and platelet-derived growth factor) [155,156].

#### The Upper - Lower Airway Relationship

The current one-airway or united airway concept is supported by anatomical links and similarities in histology, pathophysiology, and immune mechanisms. Approximately 40% of patients with CRS have asthma [157,158] and many more demonstrate bronchial hyperreactivity without overt symptoms, supporting a clinical link between these 2 conditions. Conversely, asthmatic patients often report the presence of upper airway disease and the frequency increases with severity of asthma. The mechanism of the relationship is unclear. Eosinophilic inflammation is a common link between these 2 diseases, which could be consistent with the theory of united airways. The systemic nature of airway inflammation is supported by data showing that immune responses within the airway are paralleled by similar immuno-inflammatory events in peripheral blood and bone marrow. Allergen provocation of either the upper or lower airway induces not only local changes but similar findings in the other airway, peripheral blood, and bone marrow [159]. Eosinophilic inflammation, airway remodeling, and cytokine patterns are similar throughout the airway. Studies have shown that increased eosinophils in blood and sputum and elevated nitric oxide levels in asthmatics correlate with the severity of sinus CT abnormalities (reflected by sinus CT scores).

### Bacteriology

**Statement 28**: Bacteriology of CRS is different from that of ABRS.

**Strength of evidence**: Moderate

**Strength of recommendation**: Strong

**Rationale**: Bacteriology of CRS is not as well understood as that of ABRS. Frequent negative cultures, high levels of *S aureus *and coagulase-negative isolates, and a questionable role of anaerobes complicate the picture of CRS. Although the presence of *S aureus *and coagulase-negative Staphylococci (CNS) have long been believed to suggest contamination, demonstration of *S aureus*-derived enterotoxin thought to participate in the development of CRS potentially implicates this agent as an important pathogen in CRS. Association of in vitro biofilm-producing capacity and poor outcomes in post-endoscopic sinus surgery (ESS) patients also support a role for these bacteria in disease pathogenesis [160,161].

Normally, the nasal vestibule is colonized with skin flora and frequently contains *S aureus*. In healthy control subjects, the middle meatus contains a mixture of skin and mucosal flora, such as CNS, diphtheroids, viridans group streptococci, *P acnes *and other anaerobes, and also contains bacteria capable of behaving as pathogens in disease settings, such as *S aureus*, *H influenzae*, and *S pneumoniae*.

The main pathogens recovered in chronic sinusitis include *S aureus*, *Enterobacteriaceae *spp, and *Pseudomonas *spp, and less commonly *S pneumoniae*, *H influenzae*, and beta hemolytic streptococci. It is thought that CNS may be pathogenic when present in large amounts, and when seen with neutrophils in the Gram stain or on histopathology.

The role of bacteria in CRS has been difficult to understand because bacteria have been cultured in only 50% of patients undergoing primary ESS [162]. Additionally, the flora recovered is different from that in ABRS, with high recovery rates of *S aureus *and *Pseudomonas aeruginosa*. The effect by which these known pathogens exert their effect is only beginning to be explained. Despite the fact that *S aureus *can be identified in 20% to 30% of nasal or sinus cultures in healthy Caucasians, *S aureus *has nevertheless been suggested to act as a pathogen in CRS with nasal polyposis, either via a superantigen-driven mechanism [163-165], interference with tissue metalloproteinase function [147], or induction of the low-affinity glucocorticoid receptor-beta [166]. *Pseudomonas aeruginosa *is a frequent colonizer of the diseased respiratory tract and it is almost ubiquitous in adult patients with cystic fibrosis (CF). Its action is via a number of toxins and proteases. *Haemophilus influenza*, a respiratory pathogen previously believed to be important mainly in acute infections, may also be involved. In a study of bacterial biofilms in CRS using CSLM with fluorescent in-situ hybridization, the principal pathogen identified was *H influenza*, despite the fact that it was not recovered in any of the simultaneously performed conventional sinus cultures [139]. However, it was also recovered in 2 of 5 of the asymptomatic control specimens, reinforcing the importance of other factors such as host susceptibility to the development and persistence of inflammation in CRS. These reports require additional confirmation.

Bacterial resistance alone cannot explain persistence of disease. Persistence of bacteria intracellularly or as bacterial biofilms may provide some answers by furnishing what seems to many as the 'missing link' between bacterial presence and inflammation in CRS. The intracellular persistence of *S aureus *has been shown to occur between exacerbations of disease in patients colonized with this agent [167]. The presence of bacterial biofilms has been demonstrated in CRS patients in several studies, and may explain negative cultures [168-170]. Arguing for a functional link between bacterial biofilms and CRS, 2 studies have reported poor outcomes in post-ESS patients harboring *S aureus *or *Pseudomonas aeruginosa *with the capacity to form a biofilm in vitro [160,171]. This was not the case for CNS, reinforcing the concept that it is not the presence of the biofilm itself but the specific pathogenic bacteria that is responsible for this phenomenon. This finding was confirmed by a separate group of investigators [171].

#### Fungi in CRS

Fungi frequently colonize the nasal airways in healthy subjects, and there have been conflicting reports of the role of fungi in CRS [172,173]. The presence of several different species of fungi in both individuals with CRS and healthy controls has been reported, with responses to *Alternaria *sp only in those individuals suffering from CRS. Large-scale placebo-controlled trials have failed to demonstrate a beneficial effect of topical irrigation with an antifungal.

Invasive fungi (eg, *Aspergillus *spp and *Zygomycetes *[*Rhizopus*, *Mucor*, *Absidia*]) can be aggressive and are more commonly seen in immunocompromised patients (eg, bone marrow transplant, diabetic, immunosuppressive agents); these are uncommon in immunocompetent hosts. Chronic invasive fungal sinusitis, a less severe disease, can be caused by *Candida *spp, *Aspergillus *spp, *Pseudallescheria boydii*, and is seen mostly in immunocompromised hosts.

#### Take Home Points

CRS is an inflammatory disease of unclear origin. Contributors may include:

• Bacterial colonization

• Bacterial biofilms

• Eosinophilic, neutrophilic, and lymphocytic infiltrations

• Upregulation of numerous Th2-associated cytokines

• Tissue remodeling (epithelial changes, increased extracellular matrix proteins, growth factors, and profibrotic cytokines)

• Atopy determines allergic versus nonallergic classification.

Bacteriology of CRS is different from that of ABRS:

• Not as well understood as that of ABRS

• The main pathogens include:

○ S aureus

○ *Enterobacteriaceae *spp

○ *Pseudomonas *spp

• Less common:

○ S pneumoniae

○ H influenzae

○ Beta hemolytic streptococci.

○ Coagulase-negative Staphylococci (CNS).

### Predisposing Factors

**Statement 29**: Environmental and physiologic factors can predispose to development or recurrence of chronic sinus disease. Gastroesophageal reflux disease (GERD) has not been shown to play a role in adults.

**Strength of evidence**: Moderate

**Strength of recommendation**: Strong

**Rationale**: Although the mechanism has not been fully explained, a high prevalence of allergic rhinitis has been documented in CRS patients. In addition, asthma co-occurs in 40% to 70% of patients with CRS [158]. Ciliary dysfunction and immune dysfunction have also been associated with CRS [135].

Physiologic factors include conditions in which mucociliary clearance is defective (due to either an abnormality of the cilia or mucus rheology), ostia patency is lost, or immune deficiency is present [174]. Key factors of sinonasal defense (cilia, mucus, ostia) may become abnormal in conditions such as allergic rhinitis, non-allergic rhinitis, atrophic rhinitis, hormonally-induced or drug-induced rhinitis, occupational rhinitis, ciliary dyskinesia, and nasal polyposis obstructing the ostia.

#### Allergy

Epidemiological data show an increased prevalence of allergic rhinitis in patients with CRS, but the role of allergy in the development of CRS remains unclear [129,175]. The theory that swelling of the nasal mucosa in allergic rhinitis at the site of the sinus ostia predisposes to mucus retention and infection and subsequent rhinosinusitis has not been confirmed. Occupational exposures may include workplace allergens (animals, foodstuffs, chemicals), irritants, and cigarette smoke. The contribution of these irritants is unclear.

#### Aspirin Sensitivity

Aspirin (acetylsalicylic acid [ASA])-exacerbated respiratory disease is an inflammatory disease with underlying asthma, nasal polyps, and sinusitis [176]. The combined presence of asthma, nasal polyps, and ASA sensitivity is termed Samter's triad. ASA intolerance is found with variable frequency and severity in patients with rhinosinusitis with or without nasal polyps. Patients with this triad generally are sensitive to all cross-reacting non-steroidal anti-inflammatory agents (eg, ibuprofen). Patients may have life-threatening asthma attacks in addition to severe recalcitrant sinusitis. Although the precise mechanism is unclear, it may be related to inhibition of an enzyme cyclooxygenase (COX) with subsequent shunting of arachidonic acid metabolism to the lipoxygenase pathway, culminating in massive leukotriene release. Other inflammatory products may also be involved.

#### Gastroesophageal Reflux Disease (GERD)

GERD is a common gastrointestinal problem possibly associated with both upper and lower airway disease. A proposed mechanism suggests that GERD causes reflux of gastric acid into the pharynx and subsequently to the nasopharynx causing inflammation of the sinus ostium leading to sinusitis [177]. Although an association between gastroesophageal reflux and sinusitis has been suggested [178], no definitive causal relationship has been shown in a well-performed controlled study in adults [179]. Due to this lack of evidence, the hypothesis that GERD contributes to sinusitis cannot be supported.

#### Cystic Fibrosis

CF is caused by mutations within the CF transmembrane conductance regulator gene leading to altered chloride transport in secretions. In addition to the viscous mucus that blocks the lungs and digestive system, CF is also characterized by inflammation, sinus blockage, and polyposis. An estimated 5% to 86% of children with CF have nasal polyposis [180]. Approximately half of CF carriers also reported CRS, suggesting an interaction between the CF gene mutation and CRS [181]. Of interest is that biopsy samples in CF show a predominantly neutrophilic infiltrate, suggesting that development of nasal polyposis may occur via a different pathogenic mechanism, however both Th1 and Th2 cytokine profiles have been reported in patients with CRS [182].

#### Take Home Points

Both environmental and physiologic factors that can predispose to/be associated with CRS:

• Allergic rhinitis

• Asthma

• Ciliary dysfunction

• Immune dysfunction

• Aspirin-exacerbated respiratory disease

• Defective mucociliary clearance

• Lost ostia patency

• Cystic fibrosis.

### Management of CRS

**Statement 30**: When diagnosis of CRS is suggested by history and objective findings, oral or topical steroids with or without antibiotics should be used for management.

**Strength of evidence**: Moderate

**Strength of recommendation**: Moderate

**Rationale**: Once a diagnosis based upon symptoms and confirmed by imaging or endoscopy is made, contributing or predisposing factors must be identified and addressed. Unless faced with a complication or severe illness putting adjacent structures or individual's overall health in jeopardy, initial treatment for individuals with CRS is medical (Figure 3). CRSsNP is managed with nasal or oral corticosteroid and oral antibiotics. In CRSwNP, topical INCS and short courses of oral steroids are the mainstay of management, with simultaneous oral antibiotic therapy indicated only in the presence of symptoms suggesting infection (eg, pain or recurrent episodes of sinusitis, or when purulence is documented on rhinoscopy/endoscopy).

Selection of antibiotic therapy differs from ABRS as bacteriology is different, and tends to be broader spectrum. Duration of therapy has not been defined but trends towards a slightly longer duration than that of ABRS.

#### Take Home Points

General management strategies for CRS:

• Identify and address contributing or predisposing factors

• Oral or topical steroids with or without antibiotics:

○ Antibiotic therapy should be broader spectrum than for ABRS

▪ Empiric therapy should target enteric Gram-negative organisms, *S aureus*, and anaerobes in addition to the most common encapsulated organisms associated with an ABRS (*S pneumoniae, H influenzae, M catarrhalis*)

○ Use antibiotics with broad-spectrum coverage (eg, amoxicillin-clavulanic acid inhibitors, fluoroquinolones such as moxifloxacin)

○ Antibiotic therapy duration tends to be slightly longer than for ABRS.

In the absence of complication or severe illness, initial treatment is medical:

• CRSsNP: nasal or oral corticosteroid and oral antibiotics

• CRSwNP: topical INCS and short courses of oral steroids

○ Simultaneous oral antibiotics indicated only in the presence of symptoms suggesting infection.

#### Adapting Therapy to Pathophysiologic Differences

##### CRS without polyps

Bacterial infections are believed to play an important role in patients with CRSsNP. When possible, cultures for bacteria and fungi should be obtained using methods to minimize nasal contamination.

Despite the frequent presence of positive bacterial cultures in CRS, INCS are of benefit and should be prescribed for all patients when diagnosis is confirmed by objective means. Maximal medical therapy consisting of antibiotics with or without a short course of oral steroids should be prescribed at the initiation of therapy. Ancillary measures such as saline irrigation may be of help. A short course of oral corticosteroid may be required for more severe symptoms or persistent disease, according physician assessment.

##### CRS with polyps

INCS remain the mainstay of therapy. These may be complemented by a short course of oral steroids in symptomatic subjects. Leukotriene receptor antagonists may warrant a clinical trial, especially in patients with ASA sensitivity. In CRSwNP, the presence of symptoms suggesting infection (eg, pain or recurrent episodes of sinusitis, or when purulence is documented on endoscopy) warrants combined therapy with empiric or culture-directed antibiotics.

#### Medical Therapy

##### Anti-infective Agents

Antibiotic therapy is considered an important component in managing exacerbations of CRS but should be combined with anti-inflammatory therapy to manage both the inflammatory and infectious components that contribute to the development and persistence of CRSsNP.

Although studies of antibiotics show their utility in the treatment of ABRS, antibiotic use in CRS is based on extension from first principles. Antibiotic selection must be judicious; however, guidance as to selection of optimal agent is still unclear and should, for the moment, be based on logic of bacterial flora. Empiric antibiotic therapy must be broader than in the treatment of ABRS because of the greater likelihood of infectious agents such as *S aureus*, Gram-negative enteric organisms, and anaerobic organisms. Thus, if appropriate bacteriologic samples cannot be obtained and empiric therapy is required, as in most clinical situations, consideration should be given to therapies that target enteric Gram-negative organisms, *S aureus*, and anaerobes in addition to the most common encapsulated organisms associated with an ABRS (*S pneumoniae, H influenzae, M catarrhalis*). Thus, amoxicillin-clavulanic acid inhibitors or fluoroquinolones (eg, moxifloxacin) may be prescribed.

There are no high-quality, large-scale, placebo-controlled studies of antibiotics for CRS. Quality evidence for the use of antibiotics in CRS is thus somewhat limited. In 2 comparator studies of short-term antibiotic use for CRS comparing amoxicillin clavulanate to ciprofloxacin [183] or cefuroxime axetil [184], clinical cure rates were 51% and 50% for amoxicillin clavulanate and ciprofloxacin, respectively, and 95% and 88% for amoxicillin clavulanate and cefuroxime. This difference in outcomes may be a reflection of the criteria used to diagnose the disease and to determine success.

A number of studies have reported that long-term (eg, 3 months) treatment with low-dose macrolides (eg, roxithromycin, clarithromycin) is effective in improving symptoms of CRS in adults [185-188]. However, these series were small, and with the exception of 1 study, did not include a placebo arm. Additionally, the mechanism of the effect is not well understood, but may be related to the ability of macrolides to inhibit the local host immune response and diminish the virulence of bacteria [19], rather than via the eradication of bacteria. Despite the potential interest of this approach to medical therapy, macrolide therapy in its current status has significant limitations and is not recommended as standard therapy in these guidelines. It is of particular interest that subgroup analysis in at least one of these studies has shown this therapy to be active only in those individuals with low serum IgE [188], suggesting that this effect might be limited to those individuals with "neutrophilic" chronic sinus disease, as opposed to the "eosinophilic" disease. However, the optimal patient phenotype for this type of therapy remains to be better defined.

The use of topical antibiotics has been studied, with results ranging from modest benefit to no benefit [189-191]. However, these studies were performed in post-ESS settings, where sinus ostia are widely patent and therapy is capable of directly penetrating the surgically-widened ostia of the sinuses. Larger, well-designed studies are needed to clarify the role of this approach to treat unoperated patients with CRS because penetration of the antibiotic into the sinus may not be optimal in the that setting.

##### Nasal Corticosteroids

The benefits of INCS in CRS are attributed to their anti-inflammatory properties and effects in relieving nasal congestion and shrinking nasal polyps. Early studies of INCS in patients with CRSwNP reported benefit in reducing polyp size and improving nasal symptoms [192-194]. Recent large-scale randomized trials have confirmed the efficacy of INCS in patients with CRSwNP [195-199]. One study reported that compared with placebo, once or twice daily mometasone furoate significantly reduced polyp grade score and improved the symptoms of congestion/obstruction, anterior rhinorrea, postnasal drip, and loss of smell [197]. Other large, double-blind studies reported significant improvements in nasal congestion/obstruction and reduced polyp size with mometasone compared with placebo [198,199]. Studies of budesonide have also been reported to produce significant reductions in polyp size and improve symptoms compared with placebo [195,196]. These therapies have been well tolerated.

Inconsistent results have been reported in patients with CRSsNP. Studies of INCS for CRSsNP are fewer in number and suffer from a lack of standardized patient definitions and trial design, making comparisons difficult. In a study of patients failing antibiotic therapy, a 20-week course of budesonide nasal spray significantly decreased nasal congestion and discharge and improved sense of smell compared with placebo [200]. However, in a small study of fluticasone propionate in patients with CRS, no benefit versus placebo was reported [201]. Larger, well-defined studies of INCS effectiveness in patients with CRSsNP are needed [202]. The safety of long-term therapy has nevertheless been documented. Long-term treatment with INCS may cause minor epistaxis, but is not associated with adverse structural changes or thinning of the epithelium [203].

Despite the absence of strong supporting evidence, given the pronounced inflammatory component in both CRSwNP and CRSsNP, it is the consideration of the group that treatment with INCS is an important part of the management of CRS and should be included in all patients with CRS with or without nasal polyps.

For severe polypoid disease not responding to INCS, studies have reported that a short course (2 weeks) of prednisone is effective to reduce polyp size, followed by long-term INCS to maintain the benefit [204-206]. Short-course systemic steroids have also been of benefit prior to endoscopic surgery. The minimal effective dose of systemic corticosteroids should be used to minimize potentially serious side effects [19].

#### Take Home Points

CRS without polyps:

• INCS should be prescribed for all patients

○ Benefits include:

▪ Addressing inflammatory component of CRS

• Antibiotics with or without a short course of oral steroids should be prescribed at the initiation of therapy

• Ancillary measures such as saline irrigation may be of help

• A short course of oral corticosteroid may be required for more severe symptoms or persistent disease.

CRS with polyps:

• INCS are the mainstay of therapy

○ Benefits include:

▪ Addressing inflammatory component of CRS

▪ Relieving nasal congestion

▪ Shrinking nasal polyps

• A short course of oral steroids may be prescribed in symptomatic subjects

○ 2-week course of prednisone may reduce polyp size in patients unresponsive to INCS

• Leukotriene receptor antagonists may warrant a trial, especially in patients with ASA sensitivity

• Combined therapy with empiric or culture-directed antibiotics are indicated in the presence of symptoms suggesting infection (eg, pain or recurrent episodes of sinusitis, or when purulence is documented on endoscopy).

##### Adjunct Therapy

**Statement 31**: Many adjunct therapies commonly used in CRS have limited evidence to support their use. Saline irrigation is an approach that has consistent evidence of benefiting symptoms of CRS.

**Strength of evidence**: Moderate

**Strength of recommendation**: Moderate

**Statement 32**. Use of mucolytics is an approach that may benefit symptoms of CRS.

**Strength of evidence**: Option

**Strength of recommendation**: Moderate

**Statement 33**. Use of antihistamines is an approach that may benefit symptoms of CRS.

**Strength of evidence**: Option

**Strength of recommendation**: Weak

**Statement 34**. Use of decongestants is an approach that may benefit symptoms of CRS.

**Strength of evidence**: Option

**Strength of recommendation**: Weak

**Statement 35**. Use of leukotriene modifiers is an approach that may benefit symptoms of CRS.

**Strength of evidence**: Weak

**Strength of recommendation**: Weak

#### Rationale

##### Saline

Buffered saline irrigation facilitates mechanical removal of mucus, decreases crusting, and is thought to facilitate removal of infective agents and inflammatory mediators, and increase ciliary beat frequency [207]. Thus, it is a valuable adjunctive therapy that is used in a variety of sino-nasal conditions ranging from CRS and allergic rhinitis to post-operative care. Although well-designed studies in CRS are lacking, a recent Cochrane review reported that nasal saline irrigation was effective in relieving symptoms of CRS [207].

##### Mucolytics

Guaifenesin has been demonstrated to be an effective expectorant and theoretically should benefit removal of tenacious mucus from sinuses. A small study of patients with human immunodeficiency virus and sinonasal disease reported less congestion and thinner postnasal drainage with guaifenesin versus placebo [116]. However, no clinical trials have evaluated the use of mucolytic agents in patients with CRS, so its use remains empiric. Recommended doses of mucolytics are high (eg, guaifenesin 2400 mg/d) [208] and are not available in these concentrations in Canada.

##### Antihistamines

No clinical trials have demonstrated that antihistamines improve symptoms in patients with CRS, but antihistamines have reported benefit in patients with documented inhalant allergies [209].

##### Decongestants

Due to the concern of aggravating CRS due to development of rhinitis medicamentosa from long-term use of topical decongestants [210], prolonged use of these agents should be avoided. Oral decongestants have not been adequately evaluated in CRS but concerns remain regarding systemic effects with long-term use. They may be of benefit during short-term exacerbations from presumed viral episodes.

##### Leukotriene modifiers

Small studies of the leukotriene modifiers, zileuton and zafirlukast, have supported a potential role for these agents in alleviating symptoms in patients with sinus symptoms and nasal polyps [211,212]. Montelukast has also reported symptomatic improvement in patients with asthma and nasal polyposis [213], as well as preventing recurrence of polyps in patients with aspirin sensitivity [214,215]. However, larger randomized studies are needed to explore patient subtypes likely to benefit from this approach. Leukotriene modifiers are not currently recommended for the treatment of CRS.

##### Anti-mycotic agents

Although antimycotic agents have been used in the treatment of invasive fungal rhinosinusitis and allergic fungal rhinosinusitis, they have not shown efficacy in treating patients with CRS with or without nasal polyps [216-219]. A recent large, randomized, placebo-controlled trial in patients with CRSsNP reported no difference in symptom improvement between intranasal amphotericin B and nasal saline irrigation [220].

##### Anti-inflammatory agents

COX-1 and COX-2 inhibitors have not been demonstrated to be effective in CRS aside from modifying associated pain. Macrolides have been shown to have anti-inflammatory properties in other respiratory conditions and limited data suggests these may be beneficial in CRS. Further studies are needed.

##### Immunomodulatory agents

Minimal data exists for use of agents such as interferon gamma [221]. At present there is no data to support the use of any specific cytokine or anti-cytokine in CRS.

##### Aspirin desensitization

In individuals with ASA sensitivity, treatment for aspirin-exacerbated respiratory disease (AERD) has included aspirin desensitization. Studies have demonstrated efficacy of a 3-day desensitization protocol followed by daily high dose ASA (usually 650 mg bid) in treating severe nasal polyposis in patients with AERD [222]. Some improvements have also been demonstrated with anti-leukotriene therapy in patients with AERD [223], but more studies are needed. Of note is that compliance to therapy is essential, as therapy must be reinitiated if more than 2 doses are missed. There are very few centers experienced in this therapy and none currently perform this in Canada, and thus this therapy is not recommended at this juncture.

#### Failure of response

**Statement 36**: Failure of response should lead to consideration of other possible contributing diagnoses such as migraine or temporomandibular joint dysfunction (TMD)

**Strength of evidence**: Option

**Strength of recommendation**: Moderate

**Rationale**: Alternate diagnoses that can be considered and may need to be distinguished from CRS include: allergic rhinitis, nonallergic rhinitis, vasomotor rhinitis, allergic fungal rhinosinusitis, invasive fungal rhinosinustis, nasal septal deformation, atypical facial pain, migraine or other headache diagnosis, TMD, and trigeminal neuralgia [9].

#### Take Home Points

Adjunct therapies may benefit symptoms of CRS:

• Approaches with consistent evidence of benefiting symptoms:

○ Saline irrigation

• Approaches with limited evidence of benefiting symptoms:

○ Mucolytics

○ Antihistamines

○ Leukotriene modifiers.

Failure of response should prompt consideration of other possible/contributing diagnoses:

• Allergic fungal rhinosinusitis

• Allergic rhinitis

• Atypical facial pain

• Invasive fungal rhinosinustis

• Migraine or other headache diagnosis

• Nasal septal deformation

• Nonallergic rhinitis

• Temporomandibular joint dysfunction (TMD)

• Trigeminal neuralgia

• Vasomotor rhinitis.

#### Surgery

**Statement 37**: Surgery is beneficial and indicated for individuals failing medical treatment.

**Strength of evidence**: Weak

**Strength of recommendation**: Moderate

**Rationale**: Surgery is reserved for patients who do not respond to medical therapy. Efficacy of surgery has not been assessed as extensively as has that of medical therapy but response rates of 50% to 90% have been documented in prospective series. Studies of the impact of ESS on patient quality of life have consistently reported significant improvement after the surgery [224,225].

Compelling clinical trial evidence for the efficacy of ESS remains scanty [226]. Trial design is possibly an issue. ESS remains a vital tool in the physician's armamentarium for clearing diseased mucosa, relieving obstruction, and restoring ventilation, but it should be reserved for those individuals having failed maximal medical therapy. The definition of maximal medical therapy remains to be standardized. Theoretical risks of surgery need to be counterbalanced with the equally important risks of prolonged courses of antibiotics and oral steroid therapy.

Among the CRS cases that prove difficult to cure utilizing medical management alone, a majority of patients have a combination of pathophysiological and anatomical factors predisposing to the chronic inflammation and bacterial presence [227]. Most of these patients will need to be referred to an otolaryngologist for assessment of disease and for maximal medical management, if not already administered. A percentage of these patients will reverse their disease without surgical intervention, however the majority will require appropriate medical management pre- and post-surgery for a successful outcome.

In CRS, the goal of surgery is to re-establish sinus drainage by removing excess tissue responsible for obstruction and bony areas in narrow areas. The extent of surgery is guided by the degree of sinus involvement. Minimally invasive ESS techniques are now used and often performed on a day surgery basis. Meta-analyses of studies of ESS for adult CRS have reported improvement in symptoms and quality of life [228], and fatigue [229] but these same analyses also note the lack of high-grade evidence.

**Statement 38**: Continued use of medical therapy post-surgery is key to success and is required for all patients. Evidence remains limited.

**Strength of evidence**: Moderate

**Strength of recommendation**: Moderate

**Rationale**: Early postoperative care varies between individual surgeons but usually involves antibiotics, topical or oral corticosteroids, and saline irrigation. Postoperative pain and dysfunction should be minimal and patients who develop severe symptoms of pain, temperature, or new-onset colored secretions should be referred rapidly back to the operating surgeon.

INCS after ESS has shown variable results. One study of patients post-ESS reported that a 3-week course with budesonide improved symptom scores and decreased inflammatory mediators in allergic patients with CRS [230]. In a 5-year study of patients following ESS, use of fluticasone propionate nasal spray twice daily significantly improved symptom and polyp scores [231]. However, another study reported that fluticasone- and placebo-treated patients had similar rates of polyp recurrence and CRS during the first year after ESS [232]. In a study of CRSwNP and CRSsNP patients post-ESS, a 6-month course of mometasone furoate improved an endoscopic combination score for inflammation, edema, and polyps, compared with placebo, particularly in patients with CRSwNP. Improvement in other endoscopic parameters did not reach statistical significance. Study authors reported that mometasone furoate improved wound healing post ESS [233]. Another study of mometasone furoate examined time to polyp relapse post ESS. In this study, patients receiving mometasone furoate 200 mcg twice daily had significantly longer time to relapse compared with placebo [234].

Systemic steroids have been used preoperatively, with benefit being reported postoperatively [235]. Nasal saline irrigation is recommended [135], although robust clinical trial data is lacking. Because CRS has been reported to recur in patients with high peripheral eosinophil counts, asthma, or mucosal eosinophil CRS, these patients should be followed closely [236], and may require long-term treatment with anti-inflammatory agents (steroids).

#### Take Home Points

Endoscopic Sinus Surgery (ESS)

• Indicated for patients who fail maximal medical therapy

• Goal:

○ Clear diseased mucosa

○ Relieve obstruction

○ Restore ventilation

• Provide specialist referral

• Provide post-surgical follow-up

○ Immediate postoperative care involves antibiotics, topical/oral corticosteroids, and saline irrigation

○ Monitor patient for severe symptoms of pain, fever, or new-onset colored secretions

▪ Immediately refer to operating surgeon

○ Continued care includes nasal saline irrigation and INCS, with limited evidence

○ CRS patients with high peripheral eosinophil counts, asthma, or mucosal eosinophil CRS should be followed closely, and may require long-term treatment with anti-inflammatory agents (steroids).

#### When to Refer

**Statement 39 Part A**: Patients should be referred by their primary care physician when failing 1 or more courses of maximal medical therapy or for more than 3 sinus infections per year.

**Strength of evidence**: Weak

**Strength of recommendation**: Moderate

**Rationale Part A**: Symptoms not responding to initial therapy require confirmation of diagnosis by endoscopy or CT scan. Endoscopic culture may help direct therapy.

**Statement 39 Part B**: Urgent consultation with the otolaryngologist should be obtained for individuals with severe symptoms of pain or swelling of the sinus areas or in immunosuppressed patients.

**Strength of evidence**: Weak

**Strength of recommendation**: Strong

**Rationale Part B**: Severe symptoms can be suggestive of incipient complications and may require urgent imaging, antibiotic therapy, and possible surgical drainage to prevent development of complications.

No improvement in symptoms after 4 weeks of maximal medical management (allergen avoidance measures, topical steroids, nasal irrigation, systemic antibiotics) or the presence of suspected orbital or neurological complications (as noted above) warrant referral to an otolaryngologist.

#### Allergy Testing

**Statement 40**: Allergy testing is recommended for individuals with CRS as potential allergens may be in their environment.

**Strength of evidence**: Option

**Strength of recommendation**: Moderate

**Rationale**: The role of allergy in CRS is not well understood. However, allergy has been reported to be present in 60% of patients with CRS refractory to medical treatment [129]. In 1 study, nearly half of patients with CRS and previous sinus surgery reported that immunotherapy was needed to address their symptoms [237]. Thus, allergy testing is useful to identify patients with allergic components of rhinosinusitis that might respond to allergy treatment (eg, avoiding environmental triggers, or taking appropriate pharmacotherapy or immunotherapy).

#### Immune Function

**Statement 41**: Assessment of immune function is not required in uncomplicated cases

**Strength of evidence**: Weak

**Strength of recommendation**: Strong

**Rationale**: Immune testing is not indicated in uncomplicated cases of CRS. However, it may be appropriate for patients with resistant CRS. Studies have reported that 22% to 55% of patients with refractory CRS had abnormal immunologic test results, most commonly IgG deficiency [238,239].

#### Take Home Points

Specialist referral:

• Referral to a specialist is warranted when a patient

○ Fails ≥ 1 course of maximal medical therapy *or*

○ Has > 3 sinus infections/year

• URGENT consultation w/otolaryngologist is required when a patient:

○ Has severe symptoms of pain/swelling of the sinus areas, or

○ Is immunosuppressed.

Testing:

• Allergy

○ Recommended to identify allergic components that might respond to allergy treatment (eg, avoiding environmental triggers, or taking appropriate pharmacotherapy or immunotherapy)

• Immune function

○ Not required in uncomplicated cases

○ May be appropriate for patients with resistant CRS.

### Prevention

**Statement 42**: Prevention measures should be discussed with patients.

**Strength of evidence**: Weak

**Strength of recommendation**: Strong

**Rationale**: Avoidance of predisposing allergic triggering factors is warranted despite lack of prospective studies in CRS. Both home and work environments should be assessed.

The focus of prevention in patients with CRS is to avoid acute exacerbations. Patients should be instructed to use proper hand-washing hygiene to minimize viral rhinosinusitis [127], avoid smoking [240], and perform saline nasal irrigation [207].

#### Take Home Points

The goal of **prevention **is to avoid acute exacerbations:

• Avoid predisposing allergic triggering factors

○ Assess both home and work environments for triggering factors

• Use proper hand-washing hygiene

• Avoid smoking

• Perform saline nasal irrigation.

## Summary

Despite national and international efforts to develop comprehensive guidelines, high-quality evidence for many rhinosinusitis recommendations remains limited or nonexistent. Although our understanding of the pathophysiology of acute rhinosinusitis (ARS) and chronic rhinosinusitis (CRS) has dramatically improved during the past decade, our understanding of the underlying mechanisms remains limited. This incomplete evidence base translates into continued difficulty with classifying the various forms of rhinosinusitis, selecting among available therapies, and developing new therapeutic options. Selecting appropriate therapy thus remains a challenge for both ARS and CRS. Professional experience and expert opinions are required to develop recommendations because of the absence of well-designed prospective clinical trials of therapeutic options. Future research must improve our understanding of rhinosinusitis and provide a strong evidence base for therapeutic recommendations.

Much remains to be settled in ARS to improve diagnostic criteria and direct treatment, including a better understanding of the timelines of pathophysiological changes during rhinovirus infection, host factors associated with the transition to bacterial infection, and the role of mucosal immunity, as well as severity of symptomatology and predictors of non-resolution/complications. The role of antibiotic therapy in ARS has been under scrutiny, which will likely lead to changes in clinical trial design. Improving objective methods of identifying ARS cases that warrant therapy will permit more rigorous and meaningful patient selection. Randomized, placebo-controlled studies should test the effectiveness of antibiotics and other treatments using direct measures of bacterial presence and viability at the beginning and end of therapy, rather than relying on symptomatic improvement. Trial designs that include identification of predictors of positive response to therapy would facilitate the development of recommendations.

Many aspects of CRS remain controversial and a better understanding of the pathophysiology, definitions, and role of causative factors will improve treatment approaches. As more information about CRS subtypes and novel therapies are discovered, large-scale, prospective, placebo-controlled trials of therapies will need to be repeated in the context of the various subtypes. Delivery methods that improve coverage of the nasal passages and/or penetration of the sinus cavities are needed. As results from studies of medical and surgical approaches increase, attempts should be made to identify the optimal therapy according to subtypes of disease and time point in the disease evolution. Post-operative medical management needs to be better recognized. Because there is a large number of patients with persistent signs and symptoms of the disease despite medical and surgical intervention, management guidelines specific to this group of patients should be developed.

The past 2 decades have seen a rapid increase in our knowledge of the pathogenesis, diagnosis, and management of ARS and CRS. It is hoped that over the next decade, study findings will expand and build upon the current foundation to bring scientific credibility and improved outcomes to our field.

## Abbreviations

AAO-HNS: American Academy of Otolaryngology-Head and Neck Surgery; AAP SCQIM: American Academy of Pediatrics Steering Committee on Quality Improvement and Management; ABRS: acute bacterial rhinosinusitis; AERD: aspirin-exacerbated respiratory disease; ARS: acute rhinosinusitis; ASA: acetylsalicylic acid (aspirin); CA-MRSA: community acquired methicillin-resistant *Staphylococcus aureus*; CF: cystic fibrosis; CI: confidence interval; CNS: coagulase-negative Staphylococci; COPD: chronic obstructive pulmonary disease; COX: cyclooxygenase; CRS: chronic rhinosinusitis; CRSsNP: chronic rhinosinusitis without nasal polyps; CRSwNP: chronic rhinosinusitis with nasal polyps; CSLM: confocal scanning laser microscopy; CSO-HNS: Canadian Society of Otolaryngology-Head and Neck Surgery; CT: computed tomography; EDMM: endoscopically-directed middle meatus; ENT: Ear, Nose, and Throat; ESS: endoscopic sinus surgery; GERD: gastroesophageal reflux disease; GRADE: Grades of Recommendation, Assessment, Development and Evaluation; IgE: immunoglobulin E; IL: interleukin; INCS: intranasal corticosteroids; LR: likelihood ratio; MRSA: methicillin-resistant *Staphylococcus aureus*; MSA: maxillary sinus aspirate; OR: odds ratio; PPV: positive predictive value; RR: risk ratio; SNOT: SinoNasal Outcome Test; TGF: transforming growth factor; Th: T helper cell; TMD: temporomandibular joint dysfunction; TMP/SMX: trimethoprim-sulfamethoxazole; URTI: upper respiratory tract infection.

## Competing interests

MD - Speakers Bureau: Merck Canada, Advisory board: GlaxoSmithKline, Merck Canada, Ethicon Surgical, Consultant: MedtronicXomed (bacterial biofilms), Research funding: Fondation Antoine Turmel, Fonds de recherche en santé du Québec, MedtronicXomed, PK - Advisory Boards: GlaxoSmithKline, Merck Canada, Talecris, CSL Behring, Research funding: GlaxoSmithKline, Merck Canada, Affexa Life Sciences, AK - Advisory Boards: Merck Canada, AstraZeneca Canada and Nycomed Canada, AC - Advisory Boards: Pfizer, AJ - Speaker: Merck Canada, Bayer, Abbott Canada, Nycomed Canada, MedtronicXomed, PS - Advisor to Merck Canada, GlaxoSmithKline, King, IW - Advisor: Abbott Canada, GlaxoSmithKline, Merck Canada, Pharmascience Inc. GE, EW, JB, PD, EL, AM, RS declare that they have no competing interests.

## Authors' contributions

MD conceived of the need for guidelines and coordinated the societies for representation. GE, PK, and EW participated in its design and coordinated and identified contributing authors. MD, GE, PK, EW, AK, JB, AC, PD, AJ, EL, AM, RS, PS, and IW participated in a full day meeting to review the guidelines format and content. MD, GE, PK, EW, AK, JB, AC, PD, AJ, EL, AM, RS, PS, and IW participated in the Delphi voting. MD, GE, PK, EW, AK, JB, AC, PD, AJ, EL, AM, RS, PS, and IW reviewed drafts and supplied revisions. MD, GE, PK, EW, RD, QH, and DL assessed quality of retrieved articles. MD, AK, JB, AC, PD, AJ, EL, AM, RS, PS, and IW provided first draft manuscripts for content. MD, GE, PK, EW, AK, JB, AC, PD, AJ, EL, AM, RS, PS, and IW provided review of drafts to final manuscript. MD, AK, JB, and AC provided revisions appropriate for the primary care community. MD, AK, AC and GE designed and refined the algorithms. MD and PE defined methodology. RD, QH, and DL provided expert content as required. All authors read and approved the final manuscript.
